# The red flour beetle *Tribolium castaneum*: A model for host-microbiome interactions

**DOI:** 10.1371/journal.pone.0239051

**Published:** 2020-10-02

**Authors:** Aparna Agarwal, Deepa Agashe

**Affiliations:** National Centre for Biological Sciences, Bangalore, India; University of Texas at Arlington, UNITED STATES

## Abstract

A large body of ongoing research focuses on understanding the mechanisms and processes underlying host-microbiome interactions, and predicting their ecological and evolutionary outcomes. To draw general conclusions about such interactions and understand how they are established, we must synthesize information from a diverse set of species. We analysed the microbiome of an important insect model–the red flour beetle *Tribolium castaneum*–which is a widespread generalist pest of stored cereals. The beetles complete their entire life cycle in flour, which thus serves multiple functions: habitat, food, and a source of microbes. We determined key factors that shape the *T*. *castaneum* microbiome, established protocols to manipulate it, and tested its consequences for host fitness. We show that the *T*. *castaneum* microbiome is derived from flour-acquired microbes, and varies as a function of (flour) resource and beetle density. Beetles gain multiple fitness benefits from their microbiome, such as higher fecundity, egg survival, and lifespan; and reduced cannibalism. In contrast, the microbiome has a limited effect on development rate, and does not enhance pathogen resistance. Importantly, the benefits are derived only from microbes in the ancestral resource (wheat flour), and not from novel resources such as finger millet, sorghum, and corn. Notably, the microbiome is not essential for beetle survival and development under any of the tested conditions. Thus, the red flour beetle is a tractable model system to understand the ecology, evolution and mechanisms of host-microbiome interactions, while closely mimicking the host species’ natural niche.

## Introduction

Most animals harbor specific microbes that alter various aspects of their biology, ranging from development to behavior and reproduction [1–3]. However, to harness host-associated microbiomes for practical applications such as improving host health [4], we need to better understand the factors that shape the establishment and maintenance of diverse host-microbiome relationships. Insects represent one of the best-studied animals in this context, with a large number of studies showing that insect-microbiome interactions are shaped by host taxonomy, host diet, and environmental factors [5–8]. Importantly, insects present a spectrum of host-microbial associations ranging from transient and neutrally assembled microbiomes [9–12], to established relationships whereby hosts use microbial symbionts to extract dietary nutrients, digest toxins, provide essential metabolic precursors, or protect from pathogens [3, 7]. To understand the factors that drive this spectrum of interaction strength, we need model systems where the host-microbiome interaction is suitably poised both for the evolution of a strong mutualism, as well as its breakdown. Additionally, we need to be able to experimentally manipulate the host as well as microbial partners to tease apart the functional and physiological basis of the association. While this is possible with a number of insect models such as flies, we also need model systems where it is feasible to more closely mimic the natural environment of the host.

Here, we present such a system: the red flour beetle *Tribolium castaneum*. Globally, this species infests a large diversity of cereal flours. However, it is best adapted to wheat flour, on which the beetles can complete egg-to-adult development within ~4–6 weeks. As such, they are easy to maintain in the laboratory, and have been used for over a century as model systems to address problems in ecology and evolutionary biology [13]. Importantly, laboratory conditions can closely mimic their “natural” niche (stored grain flour), rendering the results relevant for natural populations. Finally, previous studies suggest that microbes may play an important role for flour beetle biology. Over 60 years ago, Van Wyk and co-workers found that autoclaving flour reduced fecundity and increased pupation time in *T*. *confusum* (which is closely related to *T*. *castaneum*); this effect was rescued by re-introducing bacteria isolated from larvae [14]. On the other hand, antibiotics had variable and weak effects on the productivity of *T*. *castaneum* populations [15]–although the results were not interpreted in the context of microbiome impacts. A recent study also showed that the *T*. *castaneum* microbiome is important for the host to mount an immune priming response (a form of immune memory) against a bacterial pathogen [16]. Together, these studies indicate that *T*. *castaneum* may derive diverse fitness benefits from its microbiome. However, the flour beetle microbiome and its consequences for host fitness have not been systematically analysed thus far.

We addressed this using a large outbred laboratory population of flour beetles. We analysed the flour beetle microbiome and tested the impact of sex, life stage, and flour resource. We also established protocols to disrupt the flour microbiome, and analysed its impact on multiple traits relevant for fitness. Our results demonstrate that although flour beetle fitness is enhanced by flour-acquired microbes, it is not an obligate relationship, and it breaks down in novel resources. Therefore, we suggest that this is an excellent model system to understand the processes and mechanisms driving host-microbiome interactions in changing habitats.

## Methods

### Beetle populations and growth conditions

We reared beetles at 33°C (±1°C) in laboratory incubators, in round airtight plastic boxes (with holes in the lids to allow airflow) containing commercially available cereal flour. In most cases, we reared beetles on whole wheat flour (henceforth “wheat” or “wheat flour”), on which beetles have highest reproductive fitness and rapid development. As novel resources, we used corn, finger millet, or sorghum flour; all of these are cultivated in India, but are typically not heavily infested by flour beetles. To remove any prior insect infestation, we froze flour packets at -80°C for at least 4 hours and thawed the flour at room temperature before using it for experiments. To test the efficacy of this method, we added 500 eggs in 50 g wheat flour, followed the freeze-thaw protocol, and observed the flour sample for 30 days. We did not see any live beetles, confirming that freezing removes prior infestation.

For all experiments, we used beetles from a large, genetically diverse laboratory stock population, generated using flour beetles collected from 12 different locations across India. To propagate stocks, we allowed 2500–3000 individuals to oviposit in fresh wheat flour for 1 week. We then removed the adults, let the progeny develop for 4–5 weeks, and transferred newly eclosed adults to fresh flour to initiate the next generation. We maintained the stock population for ~10–12 generations in the laboratory before starting experiments.

### Manipulating the flour microbiome

To minimize contamination while handling flour and insects, we sterilized all equipment and tools (spoons, Petri dishes, brushes, tissue paper and containers) by autoclaving, wiping or flaming with 70% ethanol, or exposing to UV radiation in a laminar flow hood as appropriate. We conducted all microbiome manipulation experiments in a laminar flow hood, and placed all containers with flour or insects inside a larger sealed airtight container (with no holes in the lid), before placing them in incubators.

To deplete the flour microbial community, we spread thin layers of flour on autoclaved plastic petri dishes (90 mm diameter), using sterilized plastic spoons. We exposed the flour to UV radiation (UV-C at ~254 nm) in a laminar airflow hood for 2 h. We weighed the flour before and after UV treatment to confirm that the treatment did not significantly alter flour moisture content (which could affect beetle fitness independently of the microbiome). Alternatively, we mixed fresh wheat flour with dry, powdered forms of three different broad-spectrum antibiotics: Ampicillin, Streptomycin, and Kanamycin (0.005% w/w each). To test the impact of varying the antibiotic dosage, we mixed fresh wheat flour with varying concentrations of Ampicillin (0.005% to 0.05% w/w). While the antibiotics should not be active in dry flour, we expected that they would be active after contact with fluids in the beetles’ body.

To minimize microbial transmission via the pupal surface, we first removed excess flour on the surface with a fine brush. We then gently cleaned the pupal surface using an autoclaved #0 nylon brush dipped first in 10% sodium hypochlorite and then in 70% ethanol. In pilot experiments, we observed high mortality and dehydration of pupae; thus, we added a washing step with ultra-pure double-distilled water between the bleach and alcohol treatments. A final wash with water eliminated residual bleach on the pupal surface, minimizing toxicity via surface absorption. We used brushes for these steps because submerging pupae in alcohol and bleach (as suggested in previous studies, e.g. [16]) caused very high pupal mortality (~60% of 400 pupae did not eclose). Brushing (instead of dipping) decreased mortality to ~30%. We used thin layers of UV-treated tissue paper to absorb excess water on the pupal surface, and transferred pupae to fresh (sterile) flour for 14 days before measuring fitness.

### Collecting flour samples and experimental beetles for microbiome analysis

To characterize the flour microbiome, after our usual freeze-thaw treatment we used UV or antibiotics to create microbe depleted treatments of wheat, finger millet, corn or sorghum flour; and collected ~0.07 g samples in Eppendorf tubes using a sterilized spoon (n = 4). To test the effect of beetle infestation on the flour microbiome, we collected ‘conditioned’ wheat flour samples from stock population boxes that had been occupied by all beetle life stages for 4 weeks (n = 2). To each sample, we added 600 μl Lysis buffer from the Promega DNA extraction kit, before proceeding with DNA extraction.

To determine the beetle microbiome across sexes and life stages, we collected individuals (adult males, adult females, larvae, or pupae) directly from stock populations maintained on wheat, ensuring <1-week age variation within each life stage (n = 3–5). To test the effect of isolation (vs. group living in the stock populations described above), we isolated eggs from stocks in wells of a 96 well plate and reared them to adulthood with ~0.4 g flour/egg, obtaining 1 week old adult females (n = 6). To determine the microbiome of beetles reared on different flour resources, we collected eggs from wheat stock populations and isolated them in the respective flour (corn, sorghum, or finger millet). From the successfully eclosed adults, we randomly chose females for microbiome analysis. To test the impact of treating flour with UV (or antibiotic) on the beetle microbiome, we obtained eggs from wheat stock populations and distributed them in wells of 96 well plates containing ~0.4 g of untreated or treated flour. We stored 1 week old adults for microbiome analysis. We placed each insect at -80°C for at least 30 min, cleaned its body surface with 70% ethanol, and stored at -80°C until DNA extraction.

### Sequencing, analysing and visualizing microbiomes

To determine the bacterial community associated with flour and individual insects, we sequenced part of the 16S rRNA gene of bacterial taxa in each sample. We extracted DNA using the Promega DNA extraction kit following manufacturers’ instructions, with the following modifications. For insect samples, we first surface-sterilised individuals by dipping them in 70% molecular grade ethanol, washed in double-distilled autoclaved water to remove residual ethanol, and then crushed the whole insect with a micropestle in 600 μL Nuclei Lysis solution (from the DNA extraction kit). To minimize protein contamination, we increased the incubation time with Proteinase K from 3 h to 12 h, and washed the pelleted DNA twice in 70% ethanol. We dissolved DNA in double-distilled autoclaved water. Next, we amplified the V3-V4 hypervariable region of the 16S rRNA gene using standard Illumina primers, and prepared sequencing libraries according to the Illumina metagenomic sequencing library preparation guide [17]. We sequenced libraries on the Illumina Miseq platform (300 bp paired-end sequencing), multiplexing ~96 uniquely barcoded samples per flow cell (each with equal loading). We obtained at least 50,000 reads per sample, although the number of reads varied across individual samples as well as sample type (S1 Fig).

To determine bacterial community composition in each sample, we analysed amplicon sequencing data using QIIME v.1 [18]. We generated tables listing each OTU (Operational Taxonomic Unit) using the default parameter values in QIIME, assigning taxonomy using the Greengenes database (using 97% sequence similarity to define each OTU). In many samples, most reads mapped to mitochondria and chloroplast from flour and beetles (S1 Fig); we removed these reads from further analysis using the QIIME command “filter_taxa_from_OTU_table.py”. To reduce noise arising from transient contaminants or rare taxa, we removed all OTUs with <20 reads from further analysis. In some samples, this resulted in almost no bacterial reads. We removed these samples from further analysis since we could not distinguish whether they were poorly colonized by bacteria, or whether we had insufficient bacterial read depth due to the preponderance of reads from plastid DNA (1 of 6 females raised on wheat, 2 of 5 females raised on sorghum, and 1 of 3 larvae raised on wheat were removed from the dataset). For similar reasons, and based on prior analyses [19], we did not rarefy total reads across samples.

To visualize changes in the abundance of dominant bacteria, we focused on the five most abundant bacterial OTUs across replicate samples of a treatment (i.e. with high mean abundance across replicates; S2 Fig shows a schematic of the process using a simplified bacterial community). These dominant OTUs comprised a large fraction of the bacterial community for a given treatment (average 64% of total bacterial reads; range 35–90%). Similarly, we also visualized variation across replicates (i.e. between individual host beetles or independent flour samples). Finally, we extracted dominant OTUs identified at higher taxonomic levels (such as family and order), rather than the strain or species level resolution (see above). We expected that visualizing the impact of treatments and resources at different taxonomic resolution would provide insights into the relevance of distinct strains vs. a broader clade of related bacteria for beetle hosts. Finally, we visualised the entire bacterial community in each sample using unconstrained clustering (Principle Co-ordinate analysis, PcoA) with the ‘pcoa’ function using Bray-Curtis distances in the R package ape v5.1 [20], and generated plots using the biplots function in the R package BiplotGUI [21]. We also used constrained clustering (ordination analysis) with the CAPdiscrim function in the BiodiversityR package [22].

### Measuring effects of the microbiome on reproductive fitness

To test the impact of flour microbes on beetle fitness, we measured reproductive fitness in multiple sets of experiments, using flour or beetles with vs. without depleted microbiomes. To obtain age-controlled mated females for reproductive fitness assays, we collected pupae from stock populations across 3 days. For the microbe-depleted treatment, some pupae were also surface sterilized, in addition to receiving UV-treated flour. We sexed pupae under the microscope and placed them in same-sex groups of 3–5 pupae for 14 days (allowing them to eclose and mature sexually), with flour. We then paired adult males and females in 1 g wheat flour for 48 h to mate; removed the male; and allowed the female to oviposit for 24 h in 5 g sifted wheat flour. Sifting through a #50 sieve removes large flour particles and facilitates identifying and counting eggs. After 24 h, we removed each female, counted eggs (measuring fecundity per female), and returned the females to their Petri dish. After 3 weeks, we counted the number of surviving offspring (measuring offspring survival) and the fraction of pupae (measuring offspring development rate). At each step, we used either sterilized or untreated flour, according to the corresponding treatment. We followed this protocol for all measurements of reproductive fitness, unless specified otherwise. Sample sizes for each experiment are noted in the respective figures.

To test the effect of competition on offspring survival and development in UV-treated flour, we controlled egg density during offspring development. We collected pupae and allowed them to eclose, mature, and mate in the appropriate flour (untreated or UV-treated wheat or sorghum) for 14 days (~200 pupae per treatment). We transferred all eclosed adults to 50 g sifted wheat flour. After 24 h of oviposition, we collected and distributed 20 eggs each in 5 g of untreated or UV-treated flour (wheat or sorghum) in sterile Petri dishes (60 mm diameter). We measured offspring survival and development rate as described above. In a separate experiment, we altered the amount of resource provided to developing offspring. We isolated eggs in microplate wells, provided each egg with either 0.2 g flour or 0.4 g of flour, and measured offspring survival and development rate.

To test the impact of microbes on female fecundity across time, we isolated individual mated females (n = 25 per resource per treatment) from stock populations in ~0.7 g sifted flour (untreated, UV-treated, or antibiotic-treated), allowed them to oviposit for a fixed period (24 h, 48 h, 96 h, or 7 days), and counted the number of eggs laid per female. Since eggs hatch in 2–3 days, for the 96 h and 7 day treatments, we included larvae in the counts (i.e. eggs + larvae). We tested 7 d fecundity only in wheat, in two independent experimental blocks. For antibiotic treatments, we only tested fecundity after 48 h of oviposition, when fecundity is highest and is not confounded by hatched eggs and small larvae.

To test the impact of microbes on offspring survival in different resources (wheat, finger millet, corn or sorghum), we isolated individual eggs in single wells in a 96 well plate with ~0.4 g of either control or UV-treated flour, and counted the proportion of surviving offspring after 3 weeks.

To test whether variation in female fecundity in normal wheat flour could be explained by individual variation in the microbiome, we allowed 24 females to oviposit in isolation (in 1.5 ml Eppendorf tubes with a hole in the lid) with ~0.7 g sifted wheat flour for 48 h. We counted the eggs, and stored each female for microbiome analysis.

### Measuring effects of the microbiome on cannibalism rate

To test whether the microbiome altered larval and adult cannibalism rates, we used 2-week old larvae or adults (obtained from stock populations) to measure egg cannibalism rate in untreated vs. UV-treated flour. We mixed 20 eggs with 1 g sifted flour in a 30 mm Petri plate, and added a single larva or adult. After 24 h, we counted the number of remaining eggs and estimated the cannibalism rate as: (Number of eggs provided—Number of surviving eggs)/ Number of eggs provided. To easily differentiate eggs from flour and improve accuracy of egg counts, we stained eggs pink using neutral red dye (1% w/w mixed in flour). This is important for measuring egg cannibalism in adult females, because females can lay new eggs during the experiment, confounding estimates of cannibalism rate. Providing dyed eggs for cannibalism allowed us to differentiate them from freshly laid (white) eggs. Thus, for adult females, we calculated cannibalism rate only for dyed eggs. For larvae, we used a second experimental block to test whether the dye alters cannibalism rates.

### Measuring effects of the microbiome on host lifespan

To test the impact of microbes on beetle lifespan, we isolated eggs from wheat stock populations in 96 well microplates with 0.25–3 g wheat flour per egg (five microplates with untreated wheat flour, total 480 eggs; and two microplates with UV-treated wheat flour, total 192 eggs). After ~25 days, 68–75 individuals had pupated in each microplate. To test the impact of a lack of microbes during adulthood (vs. throughout life), we moved pupae from two microplates with untreated flour into a fresh plate with UV-treated flour. To test for effects of moving pupae into fresh flour at this stage, we moved pupae from one microplate into fresh untreated flour. Thus, we had a total of four treatments, with individuals reared (1) entirely in untreated wheat flour (“control”) (2) in untreated wheat flour until pupation, and then moved to untreated flour (3) in untreated flour until pupation and then moved to UV-treated flour, and (4) entirely in UV-treated wheat flour (“UV”). We monitored the number of surviving adults in each plate every day for 80 days after eclosion, providing fresh flour every 7 days. We repeated the experiment in a second block, with 2 microplates (n = 192 eggs) each for individuals reared entirely in untreated or UV-treated wheat flour.

### Measuring effects of the microbiome on pathogen resistance

To determine the impact of the microbiome on survival under pathogen attack, we isolated eggs, and let them develop for 2 weeks in either UV-treated or untreated wheat flour. We infected each larva with either insect Ringer solution (7.5 g NaCl, 0.35 g KCl, 0.21 g CaCl2 per L water; “sham infection”), or live culture of *Bacillus thuringiensis* (an insect pathogen; “infection”), following previously described protocols [23]. We monitored larvae every 12 h until they either pupated or died.

### Statistical analyses

We conducted all statistical analyses in R version 3.2.2 [24]. To compare the relative abundance of a specific OTU across sample groups (e.g. fresh vs. conditioned wheat flour), we used a proportion test with a continuity correction. To compare full bacterial communities across sample groups, we conducted PERMANOVA analyses using the ADONIS function in the R package ‘Vegan’ [25]. To compare microbiomes of females with varying fecundity, we binned females into three fecundity classes (0–15, 15–30, and >30 eggs per female), and used PERMANOVA analysis to test for differences in composition across classes. We compared beetle fecundity across treatments using ANOVA followed by pairwise Tukey’s HSD tests (accounting for multiple comparisons) where applicable. When data were not normally distributed, we used non-parametric Kruskal-Wallis tests. To compare survival, development rate, and cannibalism rates across treatment groups, we used generalised linear models (GLMs) with binomial errors. To compare survival count data between control (untreated) and microbe depleted flour (treated with UV or antibiotics), we used Chi-Square tests. Finally, to compare adult and larval survival across treatments, we used the Kaplan-Meir estimator to fit survival curves using the ‘survfit’ function in the R package ‘Survival’ [26].

## Results

### The beetle microbiome mirrors the flour microbiome across life stages, but varies across density and flour resource

**Fig 1:**
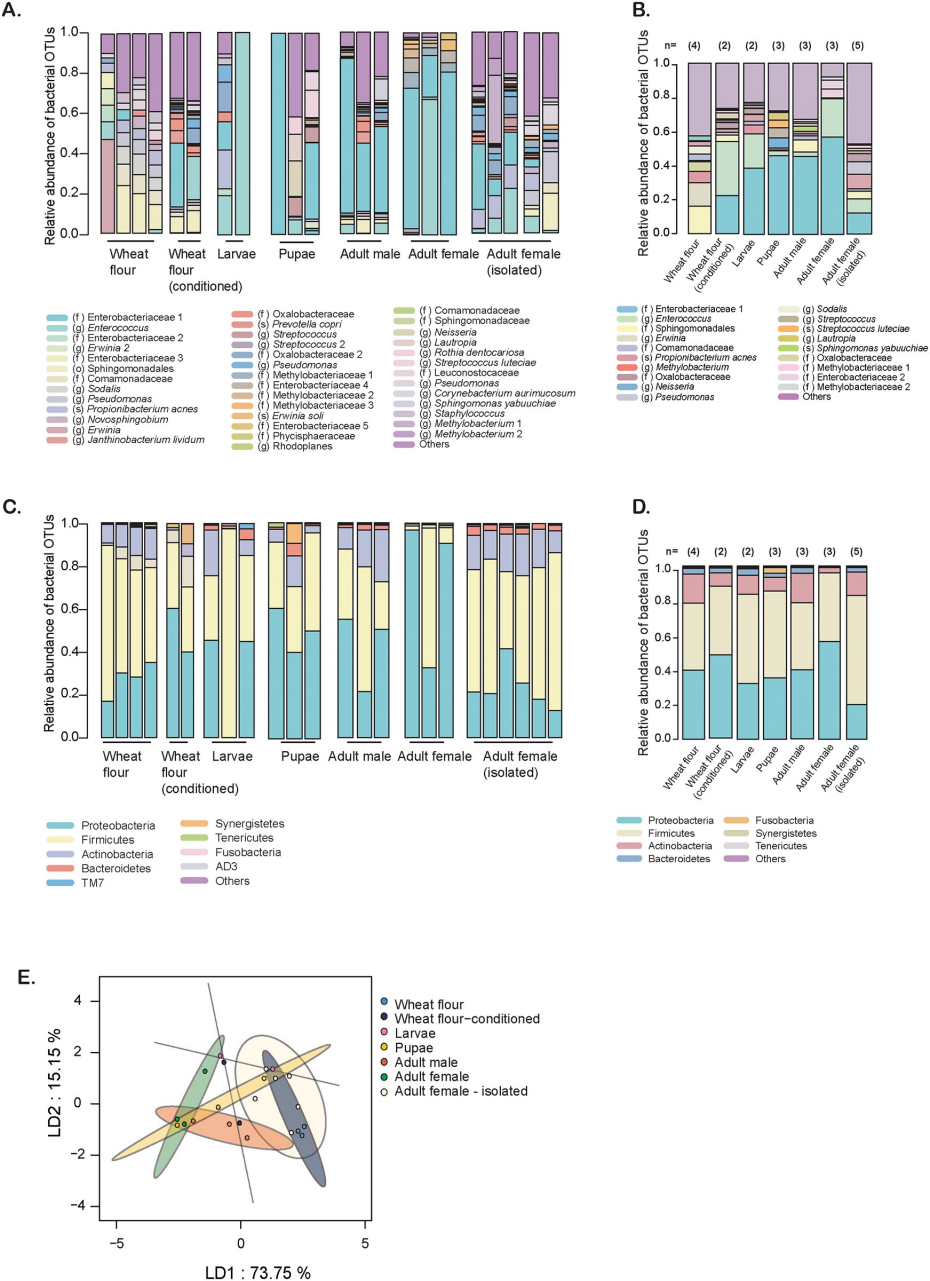
Microbiome of wheat flour and beetles reared in wheat flour. (A–D) Stacked bar plots show the relative abundance of the 5 most abundant bacterial OTUs, classified to (A, B) the lowest possible taxonomic level (see color key; o = order, f = family, g = genus, s = species) or (C, D) phylum. Panels A and C show data for individual samples of flour and beetles at various life stages; panels B and D show average relative abundance across replicates of each group (sample size is noted above each bar). In the key, numbers after each name distinguish OTUs with the same taxonomic classification (with 97% sequence identity). (E) Linear Discriminant (LD) analysis of the complete bacterial communities of flour and beetle samples; axis labels indicate % variation explained. For statistical analysis of full bacterial communities, see Table 1.

We first analysed the microbiome of wheat flour and flour beetles reared in wheat flour. On average, flour samples had ~300 bacterial OTUs (Operational Taxonomic Units) and beetle samples had ~450 bacterial OTUs. Notably, fresh wheat flour had distinct dominant OTUs from conditioned flour (used by beetles for weeks), which was in turn more similar to the dominant taxa in beetles (Fig 1A for individual samples; Fig 1B for averages). The most dominant taxa in wheat flour samples belonged to the family Sphingomonadales and the family Enterobacteriaceae (genus *Erwinia*). However, in conditioned flour as well as beetle samples, a different OTU from the family Enterobacteriaceae was dominant (“Enterobacteriaceae 1” in Fig 1A and 1B, with ~99% 16S rRNA amplicon sequence identity to bacteria from the genera *Shigella* and *Escherichia*; proportion tests, fresh vs. conditioned wheat flour: mean 0 vs. 0.22, p<0.001, fresh flour vs. all beetle samples: mean 0 vs. 0.39, p<0.001). In addition, an OTU from the genus *Enterococcus* (~99% identity to *Enterococcus faecalis*) was also much more dominant in conditioned flour and beetle samples (proportion tests, fresh vs. conditioned wheat flour: 0.03 vs. 0.32, p<0.001, fresh flour vs. all beetle samples: mean 0.03 vs. 0.22, p<0.001). Interestingly, these two OTUs were less abundant in females reared in isolation (compared to females reared in a group, in stocks; proportion tests: Enterobacteriaceae 1, p<0.001, *Enterococcus*, p = 0.01). Visualizing OTUs at a higher taxonomic level (Phylum level; Fig 1C and 1D), over 80% of the flour and beetle microbiome was dominated by Proteobacteria and Firmicutes, with greater Proteobacterial abundance in conditioned flour and female beetles from stocks.

The differences in dominant taxa were borne out by PERMANOVA analyses of the full bacterial communities. Although fresh flour tended to differ from both conditioned flour and the adult beetle microbiome (p = 0.057 and p = 0.036 respectively), conditioned flour and beetles had similar microbiomes (p = 0.1; Table 1). Bacterial communities were similar across life stages (p = 0.18) and sexes (p = 0.1) (Table 1), although our sample size (Fig 1) may limit the ability to detect small differences. Finally, the bacterial communities of females reared in isolation differed significantly from those of females reared in stock populations (p = 0.02, Table 1; these experiments were only conducted with females). These differences at the community level are readily visualized using ordination analyses (Fig 1E). Together, these results suggested that the shared flour habitat facilitates the horizontal transmission and colonization of microbes across different life stages, potentially via gut bacteria shed in beetle feces.

**Table 1:** Summary of PERMANOVA analyses comparing bacterial communities across groups.

Flour	Sample	Effect / comparison	df	Pseudo (F)	R^2^	p	Fig
Wheat	Flour	**Fresh vs. conditioned flour**	**1**	**3.0349**	**0.377**	**0.05714**	1
	Flour, beetles	**Fresh flour vs. adult beetles**	**1**	**2.7872**	**0.28**	**0.036**	1
	Flour, beetles	Conditioned flour vs. beetles	1	3.0048	0.50	0.1	1
	Beetles	Life stage	2	1.424	0.32	0.182	1
	Beetles	Sex	1	1.9463	0.28	0.114	1
	Adult females	**Isolated vs. group**	**1**	**2.2085**	**0.22**	**0.019**	1
All	Flour	**Resource**	**3**	**5.4449**	**0.31**	**9.9e-05**	2
		**UV**	**1**	**3.8374**	**0.07**	**2e-04**	4
		**Resource x UV**	**3**	**2.6233**	**0.15**	**2e-04**	4
All	Beetles	**Resource**	**3**	**2.1109**	**0.17**	**0.0032**	3
		UV	1	1.8280	0.05	0.0678	5
		**Resource x UV**	**3**	**2.4543**	**0.20**	**0.0003**	5
Wheat	Beetles	UV	1	1.6252	0.15	0.12	5
Finger millet	Beetles	UV	1	2.4305	0.45	0.1	5
Corn	Beetles	UV	1	0.4514	0.10	0.9	5
Sorghum	Beetles	**UV**	**1**	**9.6846**	**0.65**	**0.03**	5
Wheat	Beetles	**Antibiotics**	**1**	**1.9192**	**0.12**	**0.014**	6

Detailed statistical output for comparisons across flour samples, and across beetles from various sources. UV = UV-treated flour. Analyses for all flour samples and all beetle samples represent a combined PERMANOVA analysis that included interaction terms (indicated in the effect / comparison column). For beetle samples, given the significant resource x UV interaction term, we further analysed the effect of UV in each resource separately. All other tests show pairwise comparisons. Significant effects are highlighted in bold.

**Fig 2:**
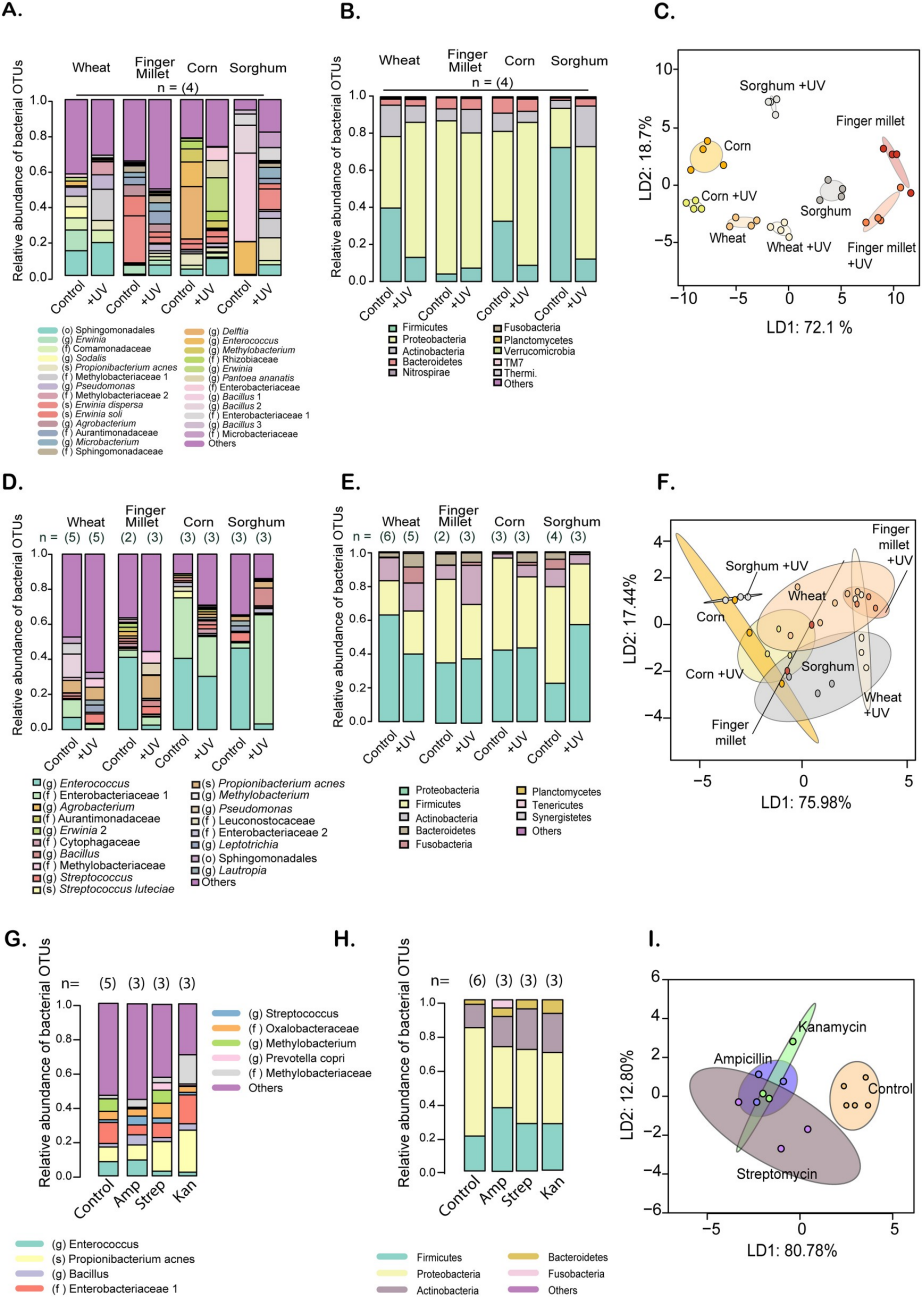
Flour and beetle microbiomes vary with flour type and bactericidal treatment. (A, B, D, E, G, H) Stacked bar plots show the average relative abundance of the 5 most abundant bacterial OTUs in flour or beetle samples, across replicates in each group (sample size is noted above bars). OTUs were classified to (A, D, G) the lowest possible taxonomic level (see color key; o = order, f = family, g = genus, s = species) or (B, E, H) phylum level. (C, F, I) Principal Coordinate Analysis (PCoA) of the complete bacterial community of samples in panels A, D and G. Axis labels indicate % variation explained. Panels A–C show data for flour samples; panels D–F show data for beetle samples (isolated females reared in each flour); panels G–I show data for beetles reared in antibiotic treated wheat flour. In the colour keys, numbers after each name distinguish OTUs with the same taxonomic classification (with 97% sequence identity). Control = untreated flour, +UV = UV-treated flour, Amp = flour + ampicillin; Strep = flour + streptomycin, Kan = flour + kanamycin. For statistical analysis of full bacterial communities, see Table 1.

Next, we tested whether beetles can maintain a stable microbiome despite consuming distinct resources. If the microbiome is environment (i.e. flour)-derived, we expected the beetle microbiome to change in resources with distinct resident flora. Indeed, different flours had distinct microbiomes (resource effect, p<0.001; compare across “control” flours in Fig 2A–2C; Table 1; S3 Fig) with different dominant OTUs (compare across “control” treatments, Fig 2A; S3 Fig for individual samples). For instance, compared to wheat flour, the finger millet microbiome was more strongly enriched in various OTUs from the genus *Erwinia*, whereas OTUs from the family Sphingomonadales were not abundant. In contrast, corn flour was strongly dominated by the genera *Enterococcus* and *Delftia*; and sorghum flour was enriched in the genera *Enterococcus* and *Bacillus*. At the phylum level, Proteobacteria were more abundant in finger millet, whereas sorghum had the opposite skew favouring Firmicutes (Fig 2B). As predicted, beetles reared on different flours for ~35 days also had distinct microbiomes (resource effect, p = 0.003, Table 1; compare across “control” treatments in Fig 2D–2F; for individual samples, see S4 Fig). Notably, beetle microbiomes on novel flours were dramatically enriched in bacteria from the genus *Enterococcus* and the Phylum Firmicutes, whereas the OTU Enterobacteriaceae 1 (which was abundant in wheat-reared females) was abundant only in females reared in corn.

Together, these results suggested that (1) beetles acquire microbes from the flour (2) a few taxa are enriched in beetles, ultimately altering the flour microbiome (likely via beetle feces) (3) this may set up a positive feedback for further enrichment of the dominant taxa via repeated passage of the flour (mixed with feces) through the beetle gut and (4) two major bacterial clades dominate both the flour and beetle microbiomes, although their relative abundance varies across flours and beetles reared on different resources.

### Treating flour with UV radiation or antibiotics disrupts the beetle microbiome

Next, we established experimental protocols to deplete or disrupt the beetle microbiome using two methods: UV treatment of flour (which should kill flour microbes, ultimately depleting the beetle microbiome), and administering antibiotics via flour. As expected, both treatments reduced the bacterial load in flour (S5 Fig; also see S1 Fig).

In all four flours tested, UV treatment significantly altered the flour microbiome (UV effect, p<0.001, Table 1), with a striking depletion in the dominant OTUs found in untreated flour (compare control vs. UV treatments for each flour, Fig 2A–2C; also see S6 Fig). Beetles reared in UV-treated flour also tended to have different microbiomes compared to beetles reared in untreated (control) flour (Fig 2D–2F; also see S7 Fig). However, the effect of rearing in UV-treated flour was significant only for sorghum, and not the other flours (resource x UV interaction, p<0.01; UV effect in sorghum, p = 0.03, all other flours, p≥0.1, Table 1). The discrepancy between the impact of UV treatment on flour vs. beetle microbiomes may arise because a few bacterial cells may have escaped the UV radiation and subsequently colonized beetles. However, this needs to be tested in further experiments. Note that with antibiotic treatment (only tested for wheat flour), beetles showed a significantly disrupted microbiome (Fig 2G and 2H; p = 0.014, Table 1; results were consistent even after removing two potential outliers: p = 0.018; also see S8 Fig). As expected, the richness of the set of dominant bacterial taxa generally appeared to increase after UV treatment (i.e. taxa were more evenly distributed; Fig 2A and 2B); but this was not observed with the broad-spectrum antibiotics. For further experiments to test the effect of disrupting the flour microbiome, we largely used UV treatment because antibiotics may generate confounding selection favoring resistant bacteria.

### Depleting the microbiome decreases beetle reproductive fitness, but only in wheat flour

**Fig 3:**
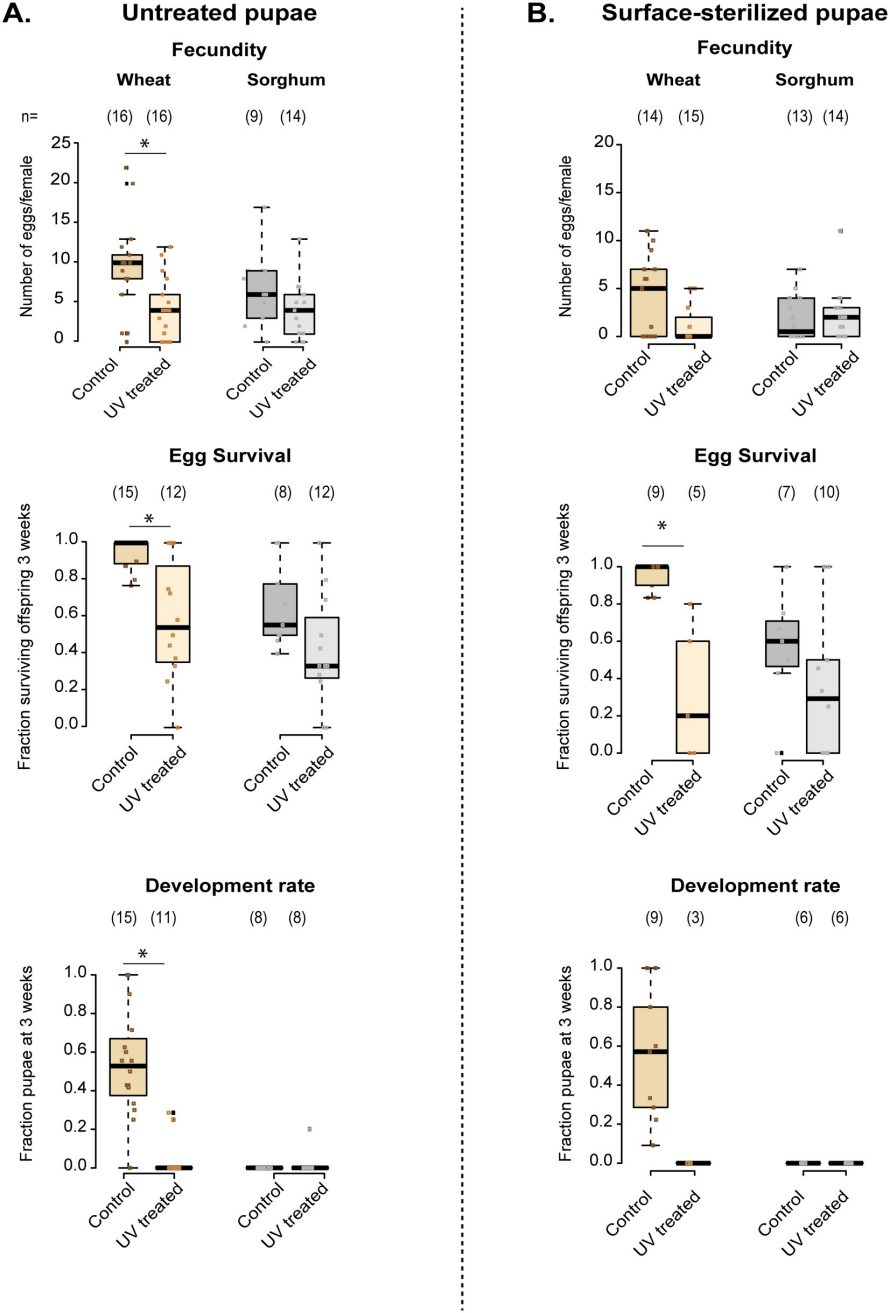
Effects of pupal surface sterilization and UV treatment of flour on fecundity, survival, and development rate. Fecundity, offspring survival and offspring development rate in untreated (“control”) vs. UV-treated wheat and sorghum, measured for adults obtained from (A) untreated and (B) surface-sterilized pupae. Boxplots show median values per female (boxes indicate interquartile length (IQL); whiskers indicate 1.5x IQL), with overlaid raw data (each point indicates data for a single female). Asterisks indicate a significant difference between control and UV treatment, based on pairwise comparisons using a generalized linear model (for full analysis with ANOVA or GLM, see S1 Table). Sample sizes are indicated in parentheses above each box. The sample sizes for offspring survival are lower because some females did not lay any eggs. Similarly, sample sizes for development rate are reduced further because replicates with no surviving offspring were removed.

We tested the impact of depleting flour microbiomes on three critical fitness-related traits: female fecundity, offspring survival, and offspring development rate. To eliminate flour-borne microbes in adults that could be transferred to eggs, we surface-sterilized pupae, allowed them to eclose without flour, and then let them mate and lay eggs. For untreated pupae, all three fitness traits decreased with UV treatment of wheat flour, but not for sorghum (Fig 3A; for full analysis with ANOVA or GLM, see S1 Table). For surface-sterilized pupae, only egg survival reduced with UV treatment in wheat flour; although the lack of an effect on development rate could arise due to the low sample size (Fig 3B; also see S1 Table). In these experiments, females laid different numbers of eggs, so offspring of each female may have experienced distinct levels of resource competition during development.

**Fig 4:**
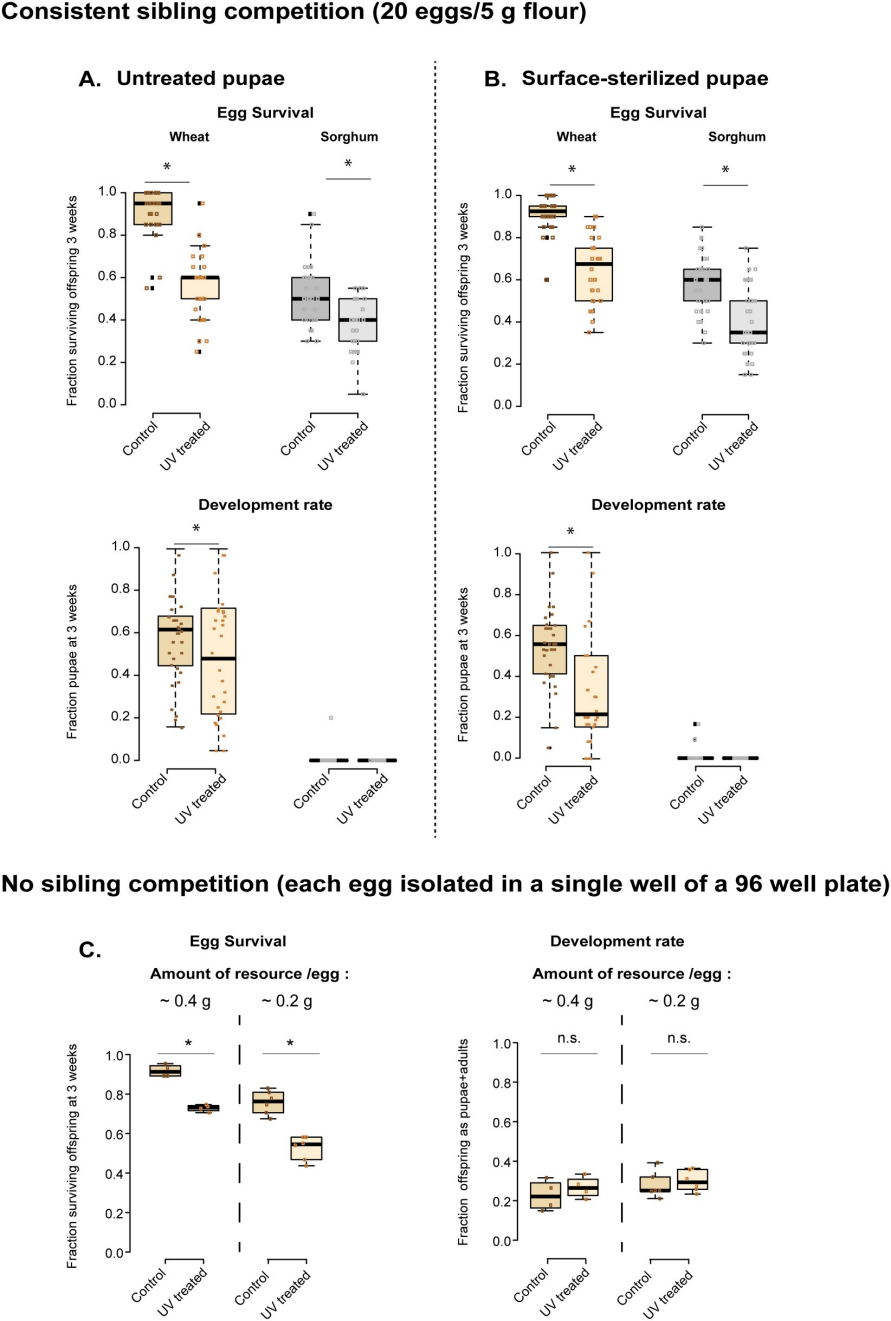
Effects of disrupting the flour microbiome on offspring survival and development rate, while controlling competition. (A–B) Offspring survival and development rate in untreated (“control”) vs. UV-treated wheat and sorghum, measured for replicate groups of 20 eggs (n = 14 replicates for sorghum; n = 15 for wheat) obtained from (A) untreated and (B) surface-sterilized pupae. (C) Survival and development rate of isolated eggs provided with either 0.2 g flour (6 microplates, 576 eggs/treatment) or 0.4 g of flour (4 microplates, 384 eggs/treatment). In all panels, boxplots show median values (boxes indicate interquartile length (IQL); whiskers indicate 1.5x IQL), with overlaid raw data (each point indicates data for one replicate). Asterisks indicate a significant difference between control and UV treatment, based on pairwise comparisons using a generalized linear model.

To test whether differential competition affected our results, we conducted another experiment where we regulated egg density (20 eggs per replicate) to maintain consistent sibling competition across replicates. Again, fewer offspring survived in UV-treated wheat; but also in sorghum, and even with pupal sterilization (Fig 4A and 4B; S2 Table). In this experiment, UV treatment also reduced offspring development rate in wheat (Fig 4A and 4B; S2 Table). Finally, when we allowed single eggs to develop with a fixed amount of resource (i.e. without sibling competition), UV-treated flour decreased survival regardless of the amount of resource (Fig 4C; S2 Table). However, with single eggs, UV treatment did not affect development rate (Fig 4C; S2 Table). Thus, the impact of UV treatment on offspring survival was robust to altered resource competition; but its effect on offspring development was sensitive to resource limitation or competition. The effect of pupal sterilization also varied across experiments. Notably, whereas pupal sterilization did not change the effect size of the UV treatment, it did reduce fecundity even in untreated flour, suggesting that pupal surface sterilization was deleterious for the treated individuals (compare fecundity in Fig 3A vs. 3B; S1 Table), though not their offspring. Hence, we did not use this method for subsequent experiments.

**Fig 5:**
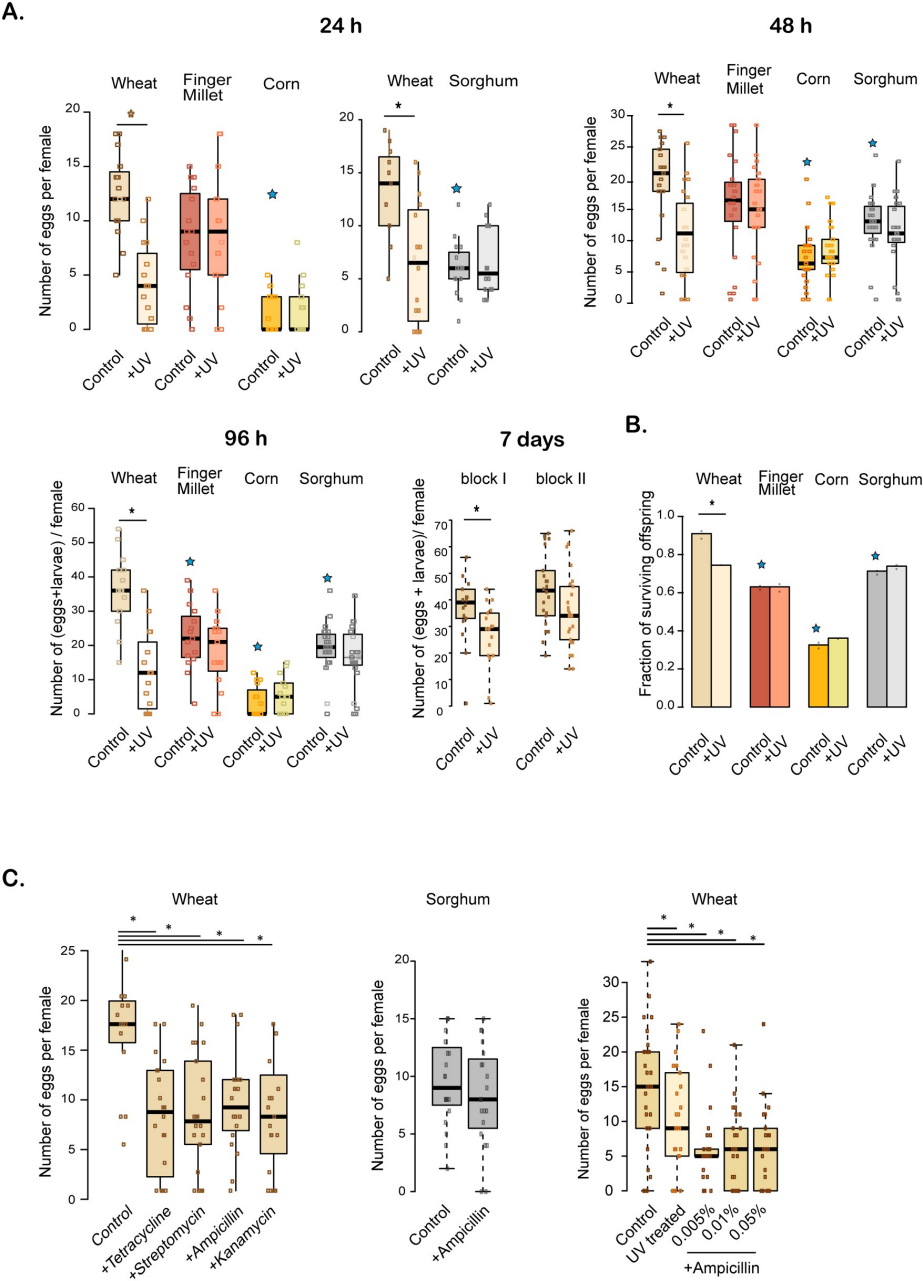
Impact of exposure to UV-treated or antibiotic-treated flour on fecundity and offspring survival. (A) Total fecundity (larvae+eggs) of females obtained from stocks and placed in untreated (“control”) vs. UV treated flour for 24 h, 48 h, 96 h or 7 d in wheat, or three novel resources (corn, finger millet, and sorghum). For the 24 h assay, sorghum was tested in an independent experimental block, with a separate wheat control; hence, it is shown as a separate figure. We included two independent experimental blocks for the 7d trial. (B) Offspring survival (average survival from eggs isolated in two 96-well plates per flour per treatment). (C) Total fecundity (larvae+eggs) of females obtained from stocks and placed in untreated (“control”) vs. antibiotic-treated flour (wheat or sorghum) for 48 hrs. In the first two panels, antibiotic concentrations are 0.005% w/w. Boxplots and raw data are as described in Fig 4; n = 25 females/treatment. Small black asterisks indicate a significant difference between control and treated resources; large coloured asterisks indicate a significant different between control for wheat vs. control for a novel resource. For statistical analysis, see S3 Table.

Finally, additional experiments corroborated the broad patterns noted above: depleting flour microbes reduced fecundity in wheat flour, but not in novel resources (corn, finger millet and sorghum), regardless of the time allowed for oviposition (Fig 5A; S3 Table). Similarly, UV treatment reduced offspring survival only in wheat flour (Fig 5B; S3 Table). Antibiotic treatment also decreased female fecundity in wheat flour but not in sorghum (Fig 5C; S3 Table). In the novel flours, in most cases larvae did not pupate in 3 weeks, so we did not measure the effect of UV treatment on development rate. Together, these experiments indicate that the beetles can acquire beneficial microbes from wheat flour, but not from novel resources.

### Female microbiome does not explain individual variation in fecundity in untreated flour

**Fig 6:**
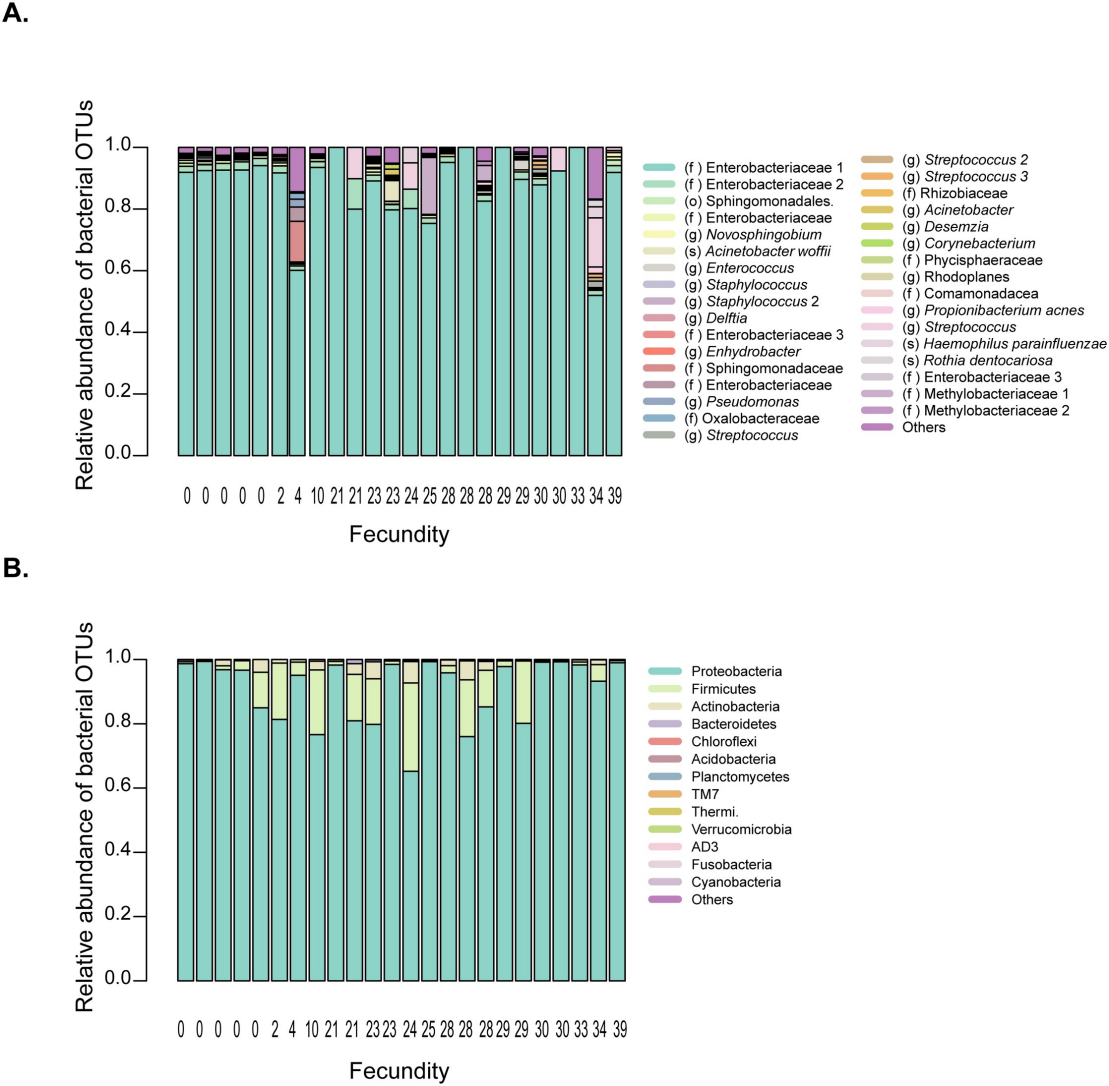
Females with varying fecundity have similar microbial communities. Stacked bar plots show the average relative abundance of the 5 most abundant bacterial OTUs, (A) classified to the lowest possible taxonomic level (o = order, f = family, g = genus, s = species). Numbers after each name distinguish OTUs with the same taxonomic classification (with 97% sequence identity). (B) Phylum level.

The strong and repeated impact of the flour microbiome (environmental microbe availability) on female fecundity prompted us to ask whether the females’ own microbiome may also influence their fecundity. Hence, we tested whether more fecund females were especially enriched in specific bacteria (e.g. potential mutualists or pathogens). However, despite wide variation in fecundity (range: 0 to 39 eggs per female), all females had a similar microbiome dominated by Proteobacteria; specifically, a single OTU belonging to the family Enterobacteriacae (Fig 6; PERMANOVA for the effect of fecundity class on microbiome composition: p = 0.2; see Methods). Thus, although females generally avoid laying eggs in microbe-depleted flour (Fig 5), individual level variation in female fecundity in normal flour is not directly driven by the female microbiome.

### Depleting the microbiome increases cannibalism rate

Cannibalism plays a major role in flour beetle biology [27–29], and cannibalism rates typically increase in poor quality resources [30, 31]. Hence, we measured egg cannibalism rates as a function of normal vs. depleted flour microbiomes. We supplied focal individuals with eggs stained with neutral red, allowing us to separate the fresh (white) eggs laid by females during the assay [32].

**Fig 7:**
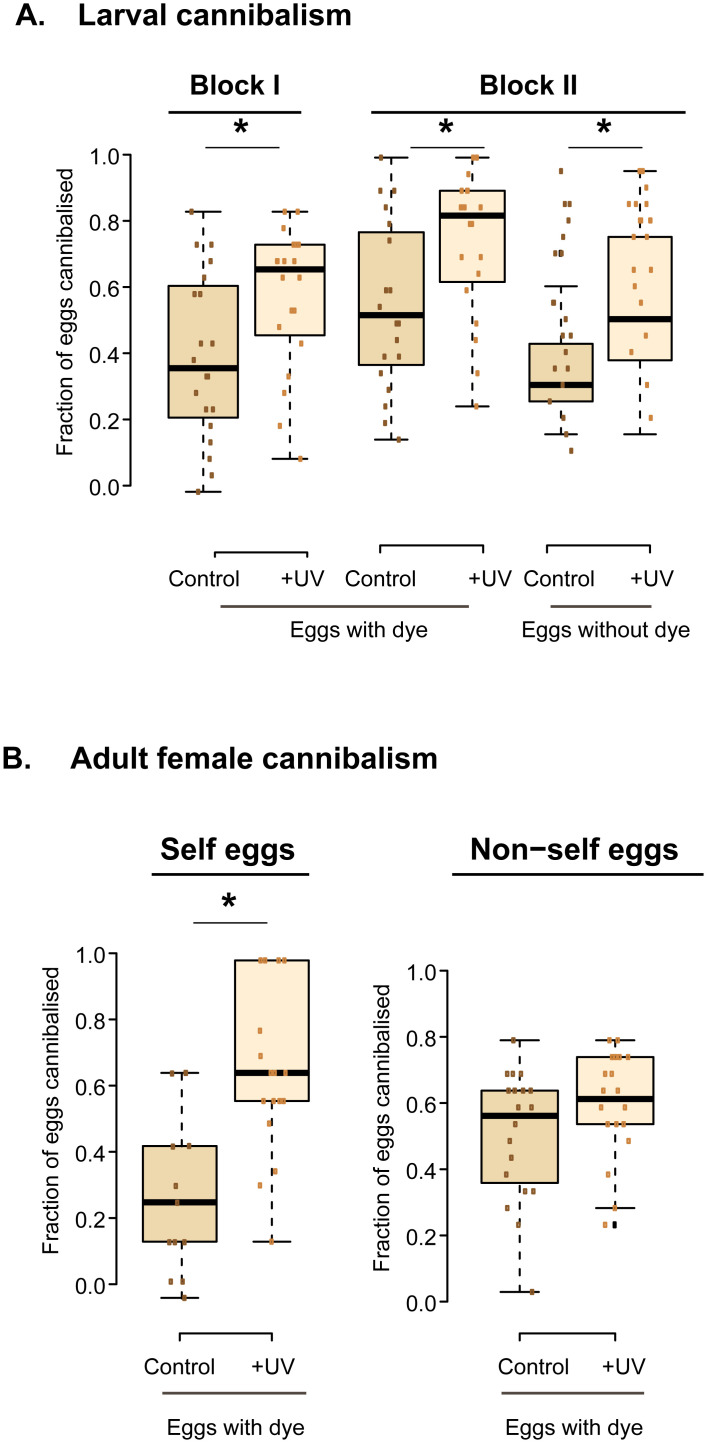
Impact of flour microbes on cannibalism rate. The fraction of eggs eaten by (A) isolated larvae (tested in two independent experimental blocks), and (B) single adult females in untreated (“control”) vs. UV treated wheat flour (n = 20 replicates/treatment). Eggs provided for cannibalism (20 eggs per replicate) were laid by females in flour with vs. without neutral red dye (which colors eggs pink). In panel B, eggs were either provided to the same female (“self eggs”) or a randomly chosen other female (“non-self eggs”). For statistical analysis, see S4 Table.

As expected, larval cannibalism increased in UV-treated flour (larvae tested in two independent experimental blocks; Fig 7A; S4 Table). Since larvae do not lay eggs, in the second experimental block we also tested whether staining with neutral red affected the cannibalism rate; and found that larvae cannibalized dyed eggs more than white eggs. However, UV and the dye did not interact significantly, indicating that UV affects cannibalism rate independent of the dye (Fig 7A; S4 Table). Female cannibalism rate also increased in UV-treated flour, but this pattern was restricted to females provided with their own eggs (laid on the previous day in flour containing neutral red; Fig 7B; S4 Table). When provided with their own eggs, females avoided cannibalism in control flour but increased it in UV-treated flour (rates increased from ~20% to ~60%). However, when provided with others’ eggs, females consumed ~60% of the eggs regardless of flour treatment. Thus, females modulate cannibalism rates in response to the environmental microbiome, as well as the provenance of available eggs.

### Depleting the microbiome reduces lifespan

**Fig 8:**
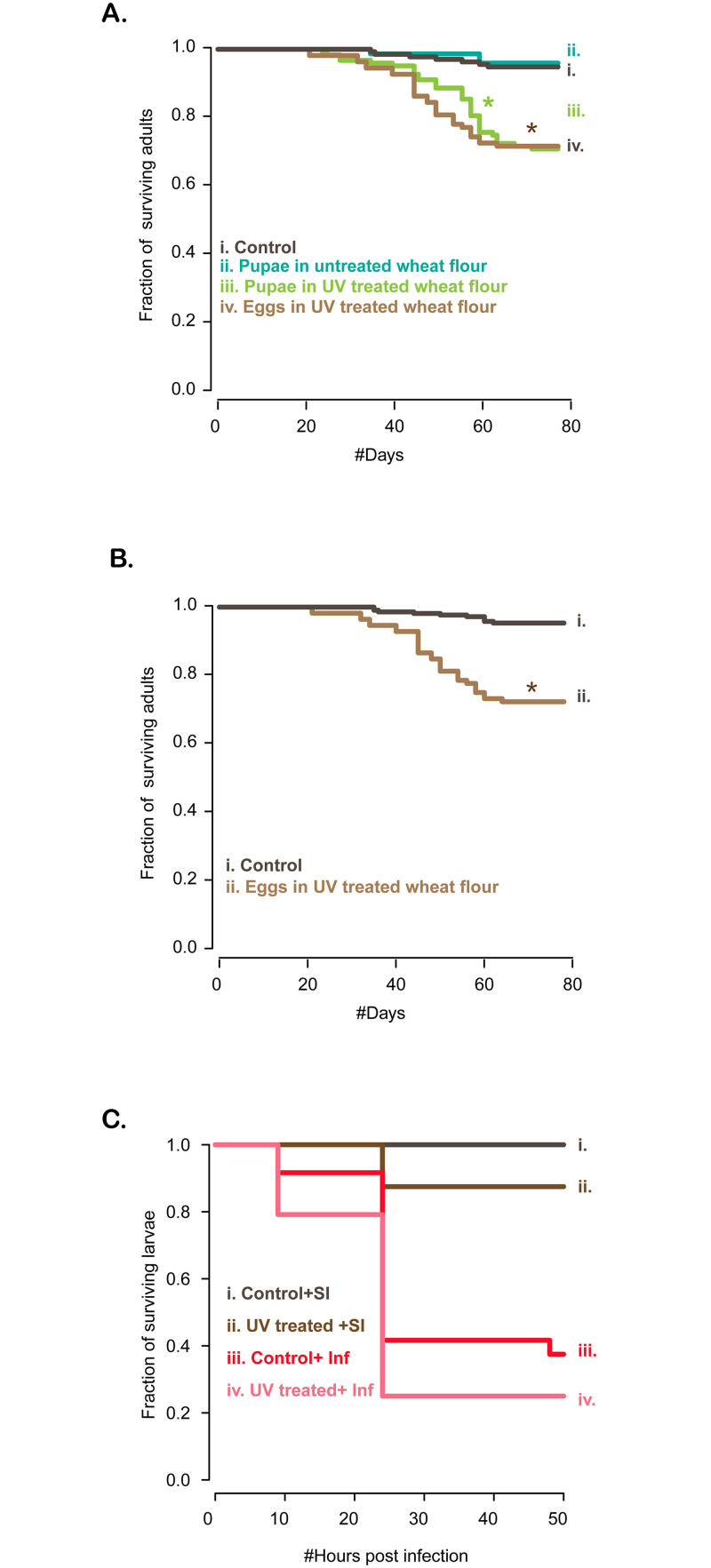
Impact of UV treatment on lifespan with and without infection. (A) The fraction of surviving adults as a function of the number of days since eclosion, for individuals isolated in wells of 96 well plates from the egg stage (n = 96–192 eggs/treatment). Control individuals (i) were reared in untreated wheat flour throughout; as pupae, some of them were either transferred to fresh untreated wheat flour (ii) or UV-treated wheat flour (iii); and eggs were reared in UV-treated wheat flour without transfer as pupae. (B) A second experimental block with two of the treatments shown in panel A (i.e. without pupal transfer) (C) Survival of larvae reared in untreated (control) or UV-treated flour, after being injected with a sham infection (“SI”) or infection with a live pathogen (“Inf”) (n = 30/treatment). For statistical analysis, see S5 Table.

Next, we tested whether an intact microbiome was important for beetle lifespan. We isolated eggs and allowed them to develop to pupation in untreated vs. UV-treated flour. To test the effect of access to flour microbes throughout development vs. only in adulthood, we split pupae from untreated flour to fresh UV-treated flour or untreated flour; and monitored survival of all pupae for ~3 months. Within two months, only ~70% of individuals in UV-treated flour survived, whereas >90% individuals from both sets of controls were alive (Fig 8A; S5 Table). The pattern was consistent even when adults had access to flour microbes until pupation, suggesting that microbes transmitted across metamorphosis are not sufficient to enhance adult fitness. These results further support the hypothesis that the beetle microbiome is continually acquired from the flour habitat. The impact of flour microbes on adult lifespan was also observed in a second experimental block (Fig 8B; S5 Table), demonstrating that depleting the microbiome significantly reduced adult beetle lifespan.

### Depleting the microbiome does not affect survival under pathogen attack

Finally, we tested whether the flour microbiome affected the ability of larvae to survive infection by a pathogen. We infected larvae either with a live culture of *Bacillus thuringiensis* (“Inf”) or a buffer (sham infection, “SI”); and reared each group on either UV-treated or untreated (control) wheat flour. As expected, sham-infected larvae had higher survival than infected larvae; and overall, larvae reared in UV-treated flour had lower survival than control larvae (Fig 8C; S5 Table). However, within each group (sham-infected or infected), UV treatment did not significantly alter survival (S5 Table). Thus, although microbial depletion generally decreases larval survival, it was not especially detrimental during pathogen attack.

## Discussion

Here, we present the first systematic characterization of the microbiome of flour beetles as well as their primary habitat and food source (flour). We find that beetles have a flour-derived microbiome that is shared across sexes and life stages—most likely via the flour—and is enriched in specific bacterial taxa. Establishing protocols to manipulate the beetle microbiome, we show that all life stages of flour beetles derive multiple fitness benefits from their wheat flour-acquired microbiome, leading to an ~2 fold increase in fecundity and offspring survival, and ~1.5 fold increase in lifespan. When reared in sibling groups (with resource competition), offspring also develop more slowly in UV-treated flour. However, when reared in isolation (without competition), all larvae develop faster, with no effect of UV treatment. In grain and flour warehouses, eggs likely do not develop in isolation; hence, we speculate that the former results are more instructive about the true impact of the microbiome on development rate. Notably, although depleting the wheat flour microbiome clearly reduces female fecundity, the fecundity of individual females in normal wheat flour was not correlated with their microbiome. In contrast to the strong consequences of the wheat flour microbiome, removing microbes from other (suboptimal) flour types did not reduce beetle fitness. Finally, we find that in the absence of an intact flour microbiome, adult beetles and larvae increase egg cannibalism. Since flour beetle cannibalism typically increases in poor resources [28, 30, 31], we suggest that the flour microbiome may either directly or indirectly signal resource quality. Pathogen resistance was the only tested fitness metric where we did not find an impact of the microbiome; although prior work suggests that the beetle microbiome is critical for the formation of immune memory against the same species of pathogen used in our assays [16]. Overall, in conditions that closely mimic its natural habitat, the flour beetle microbiome appears to have pervasive effects on host fitness.

Despite these overarching impacts, the microbiome does not appear to be essential for any of the fitness parameters that we measured, suggesting that the host-microbiome interaction is not an obligate association. The facultative nature of the relationship (despite the potential for strong positive selection favoring the association) suggests that it may be recently established. Alternatively, the flexible nature of the relationship may itself be selectively favoured due to environmental heterogeneity. For instance, if flour beetles experience substantial spatial or temporal habitat heterogeneity and cannot reliably access appropriate microbes, the ability to maintain relatively high reproductive fitness without microbes may be beneficial. This is especially relevant for generalist species such as *T*. *castaneum*, which likely sample diverse resources even within a single generation. The lack of microbiome impacts on fitness in three different novel resources supports this hypothesis. In these resources (used commonly in India), the beetles would encounter distinct flour microbiomes compared to wheat. Further experiments are necessary to test these ideas, and to determine factors that can facilitate the evolution of a strong obligate host-microbe association akin to that observed in many other insects.

In contrast to other insect species [33, 34], we also did not find evidence for significant transmission of microbiomes from females to eggs—individuals reared from the egg stage in different flours had distinct microbiomes, instead of wheat-associated microbiomes. As discussed above, this might indicate that the association is still in the early stages of evolution. On the other hand, selection for vertical transmission may be very weak in this system because different life stages share the growth habitat and resource, allowing rapid and easy transmission of microbiomes across individuals. Such a horizontal spread of environmental microbes is likely to occur in other organisms where different life stages share resources and do not disperse away. Thus, our observations with flour beetles could perhaps be broadly generalized for other species with similar life histories. Whether environmental sharing of the microbiome strengthens or hinders subsequent evolution of host-microbial associations remains to be tested.

Interestingly, we found that the flour beetle microbiome varies across individuals, with some individuals showing almost no bacterial reads. We speculate that this is likely an artifact of the large fraction of reads from chloroplast and mitochondria—largely derived from cereal flour—and may not necessarily reflect biological differences. Due to the resulting paucity of bacterial reads (and for other logistical reasons), some of our treatments also had low sample sizes, weakening some of our conclusions. Nonetheless, the individual variation is also biologically interesting, and is not uncommon in other insects [6]. All else being equal, individuals should sample a similar microbiome, since beetle movement in flour generates a well-mixed habitat where feces as well as microbes should be evenly spread. Hence, the large variation in the microbiome across individuals suggests poor bacterial colonization of the gut. If bacteria are continually flushed out of the gut, they can only be replenished via food; and variation in microbial loads across individuals may simply reflect variation in the timing of when they last consumed flour. Alternatively, variation across individuals may also reflect host genotype- or condition- dependent bacterial colonization [35, 36]. We hope that future experiments can distinguish between these hypotheses and determine the cause of large individual variation in the microbiome.

Despite the substantial individual variation in bacterial OTUs, the dominant bacterial phyla were largely consistent across hosts, suggesting that host reliance on bacteria is fairly general at higher levels of bacterial taxonomy. The major bacterial phyla that we observed—Proteobacteria and Firmicutes—also dominate the microbiomes of most other insects, accounting for ~64% and ~7% of bacterial communities [6]. Within these phyla, bacteria from the family Enterobacteriaceae were most common and dominant bacterial taxa across flour beetles and in wheat flour; whereas bacteria from the genus *Enterococcus* were enriched in beetles reared on new suboptimal flours. Hence, we speculate that one or both of these taxa may explain a large fraction of the microbiome’s fitness impact on host beetles. The genus *Enterococcus* includes species that frequently live in invertebrate guts and are facultative pathogens that use various mechanisms such as secreted toxins, quorum sensing and cell surface adhesion to cause host damage [37]. Thus, we speculate that the specific *Enterococcus* OTU that we observed in beetles may be harmful, especially in high abundance, and in the absence of other beneficial OTUs. On the other hand, the family Enterobacteriaceae encompasses a large and very diverse set of rod-shaped bacteria, including well-known animal commensals and pathogens such as *Escherichia coli* and *Salmonella*, as well as obligate or facultative insect mutualists (e.g. in the genera *Buchnera* and *Sodalis*). Many members of this family produce toxins called microcins [38], which may explain their ability to prevent mammalian gut colonization by pathogens [39, 40]. In addition, they could directly benefit hosts by degrading complex dietary polysaccharides, or synthesizing limiting nutrients such as vitamins for the host. Unfortunately, prior studies have not examined their role in insects, so we can only speculate about the mechanisms underlying the potential fitness benefits of the OTU “Enterobacteriaceae 1” for flour beetles. Further experiments are necessary to test its specific impact on beetle physiology and fitness.

Our work identifies some avenues for further refining methods to manipulate the microbial communities of flour and flour beetles. First, the relatively weak effects of UV treatment and antibiotics on bacterial community composition may reflect the fact that neither treatment is foolproof, allowing some bacteria to persist. Therefore, to completely eliminate the beetle microbiome, stronger doses or exposure times may be necessary. Second, the flour beetle system appears to be especially prone to problems with amplification of chloroplast and mitochondrial DNA. The resulting problem of insufficient bacterial reads could potentially be resolved by using methods to reduce amplification from mitochondrial and chloroplast DNA (e.g. [41]), or by increasing total sequencing depth. The efficiency of these methods needs to be explicitly tested. Finally, it would be valuable to modify our protocols and develop techniques that can allow longer-term maintenance of germ-free beetle populations.

In summary, our work showcases flour beetles as a tractable model system allowing manipulation of host and microbial genotype, microbial community composition, microbial transmission and its effects, and analysis of the impacts of the association on host and microbial community members alike. Thus, we suggest that flour beetles are an excellent model to understand the evolution, establishment, and maintenance of host-microbial associations; especially in the context of animal guts, where such associations have important ecological and evolutionary impacts [3]. Such an understanding may also help to identify novel pest control strategies for this major global pest of stored grains.

## Supporting information

S1 Fig Number of reads obtained for all samples. Boxplots show the total number of reads and reads mapping to mitochondria and chloroplasts, for (A) Flour samples (B) Flour and beetle samples (C) Beetles reared on untreated vs. UV-treated flour (D) Beetles reared on flour mixed with antibiotics.
(TIF)

S2 Fig Schematic showing the protocol to identify the top 5 abundant bacterial OTUs across replicate samples in each treatment group. (TIF)

S3 Fig Microbiomes of different flours. (A, C) Relative abundance of the top five dominant bacterial OTUs in individual samples of flour, classified to (A) the lowest possible taxonomic level or (C) phylum level. (B, D) Average abundance across replicates shown in panels A and C. (E) Linear Discriminant (LD) analysis and (F) Principal Coordinate Analysis (PCoA) of the complete bacterial community of flour samples; axes labels indicate % variation explained.
(TIF)

S4 Fig The microbiome of beetles reared in different flours. (A, C) Relative abundance of the top five dominant bacterial OTUs in individual beetle samples (isolated adult females), classified to (A) the lowest possible taxonomic level or (C) phylum level. (B, D) Average abundance across replicates shown in panels A and C. (E) Linear Discriminant (LD) analysis and (F) Principal Coordinate Analysis (PCoA) of the complete bacterial community of beetle samples; axes labels indicate % variation explained.
(TIF)

S5 Fig Reduction in bacterial load after UV and antibiotic treatment of flour. Flour samples plated onto nutrient agar plates.
(TIF)

S6 Fig Impact of UV treatment on the flour microbiome. (A, B) Relative abundance of the top five dominant bacterial OTUs in individual samples of flour, classified to (A) the lowest possible taxonomic level or (B) phylum level. (C) Principal Coordinate Analysis (PCoA) of the complete bacterial community of flour samples; axes labels indicate % variation explained.
(TIF)

S7 Fig Impact of UV treatment of flour on the beetle microbiome. (A, B) Relative abundance of the top five dominant bacterial OTUs in beetle samples (isolated adult females reared on control vs. UV-treated flour), classified to (A) the lowest possible taxonomic level or (B) phylum level. (C) Principal Coordinate Analysis (PCoA) of the complete bacterial community of beetle samples; axes labels indicate % variation explained.
(TIF)

S8 Fig Impact of antibiotics on beetle microbiome. (A, B) Relative abundance of the top five dominant bacterial OTUs in beetle samples (isolated adult females reared on control vs. antibiotic-treated flour), classified to (A) the lowest possible taxonomic level or (B) phylum level. (C) Principal Coordinate Analysis (PCoA) of the complete bacterial community of beetle samples; axes labels indicate % variation explained.
(TIF)

S1 Table Summary statistics for results shown in Fig 3. The table shows detailed statistics for comparisons of fecundity, survival, and development rate in control (untreated) or UV-treated flour (UV), with or without surface sterilization of pupae, and varying egg density during survival and development (depending on female fecundity). SS = Surface-sterilised pupae; Untreated = not surface sterilized; Conf. = confidence interval. The model specified for each analysis is indicated. Significant effects are highlighted in bold.
(DOCX)

S2 Table Summary statistics for results shown in Fig 4. (I) The table shows detailed statistics for comparisons of egg survival and development rate in control (untreated) or UV-treated flour (UV), with or without surface sterilization of pupae; and with a fixed egg density (20 eggs per replicate). SS = Surface-sterilised pupae. The model specified for each analysis is indicated. (II) The table shows detailed statistics for comparisons of egg survival in control (untreated) or UV-treated flour (UV). Significant effects are highlighted in bold.
(DOCX)

S3 Table Summary statistics for results shown in Fig 5. The table shows detailed statistics for comparisons of fecundity in control (untreated) or microbe-depleted flour (via UV treatment or antibiotics with the indicated concentration). Conf. = confidence interval; Amp = ampicillin. The model specified for each analysis is indicated. Significant effects are highlighted in bold.
(DOCX)

S4 Table Summary statistics for results shown in Fig 7. The table shows detailed statistics for comparisons of cannibalism rates of larvae and adult females in control (untreated) or UV-treated flour (UV). Eggs were either dyed or not; and females were provided with their own eggs (self eggs) or another female’s eggs (non-self eggs) to cannibalise. The model specified for each analysis is indicated. Significant effects are highlighted in bold.
(DOCX)

S5 Table Summary statistics for results shown in Fig 8. The table shows detailed statistics for comparisons of survival rates of beetles reared in untreated wheat flour (control) or UV-treated flour (UV). As pupae, some control individuals were transferred to either fresh untreated flour ((ii) in Fig 8A) or UV-treated flour ((iii) in Fig 8A). In the table, effects are indicated as shown in Fig 8. Significant effects are highlighted in bold.
(DOCX)

S1 Dataset (XLSX)
