# Supplementary figures and images for: The red flour beetle Tribolium castaneum: A model for host-microbiome interactions
Source: PLoS One. 2020 Oct 2;15(10):e0239051. doi: 10.1371/journal.pone.0239051 (PMC7531845; doi:10.1371/journal.pone.0239051)

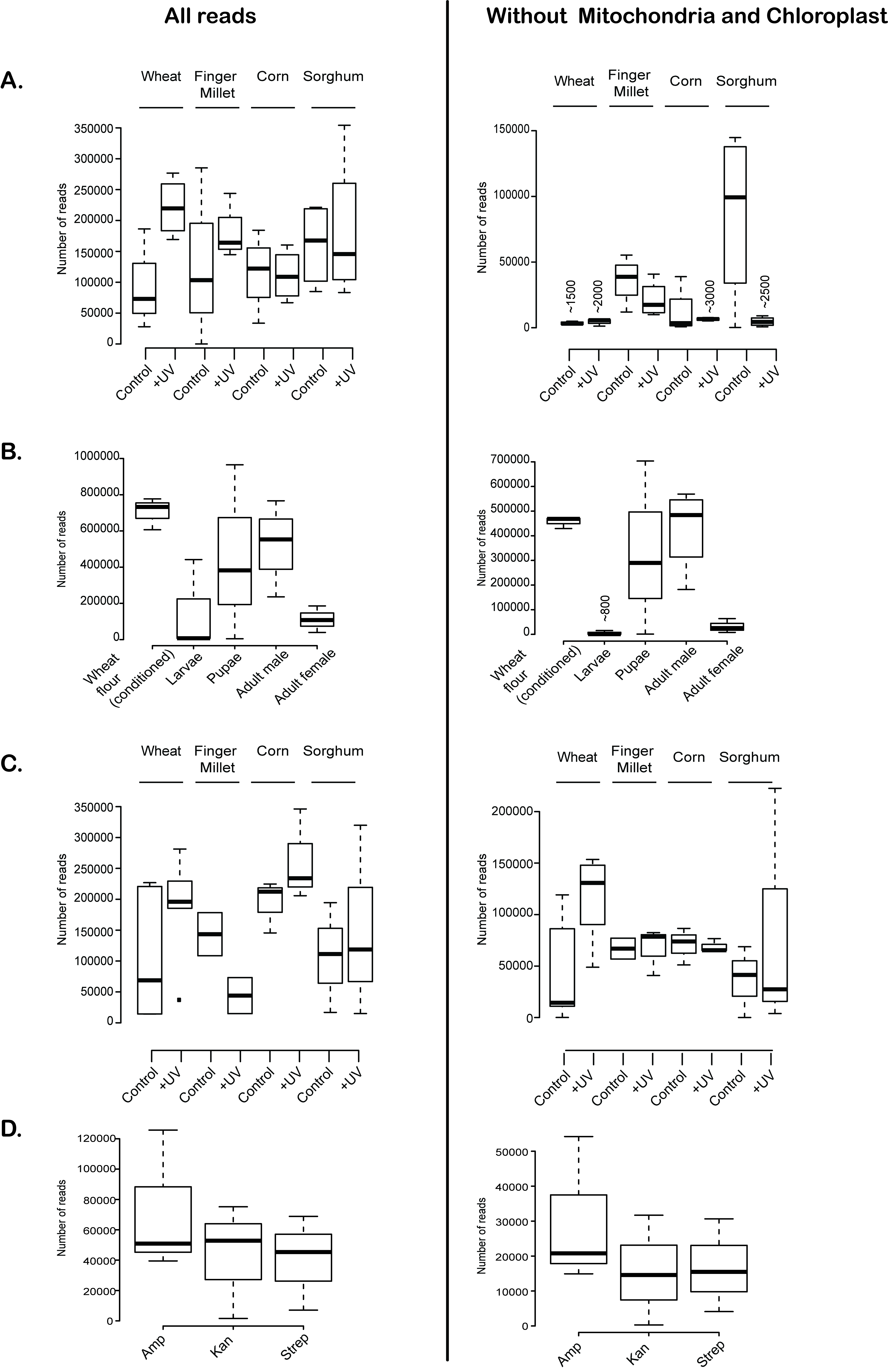

Supplement: S1 Fig — Boxplots show the total number of reads and reads mapping to mitochondria and chloroplasts, for (A) Flour samples (B) Flour and beetle samples (C) Beetles reared on untreated vs. UV-treated flour (D) Beetles reared on flour mixed with antibiotics. (TIF) [file pone.0239051.s001.tif]

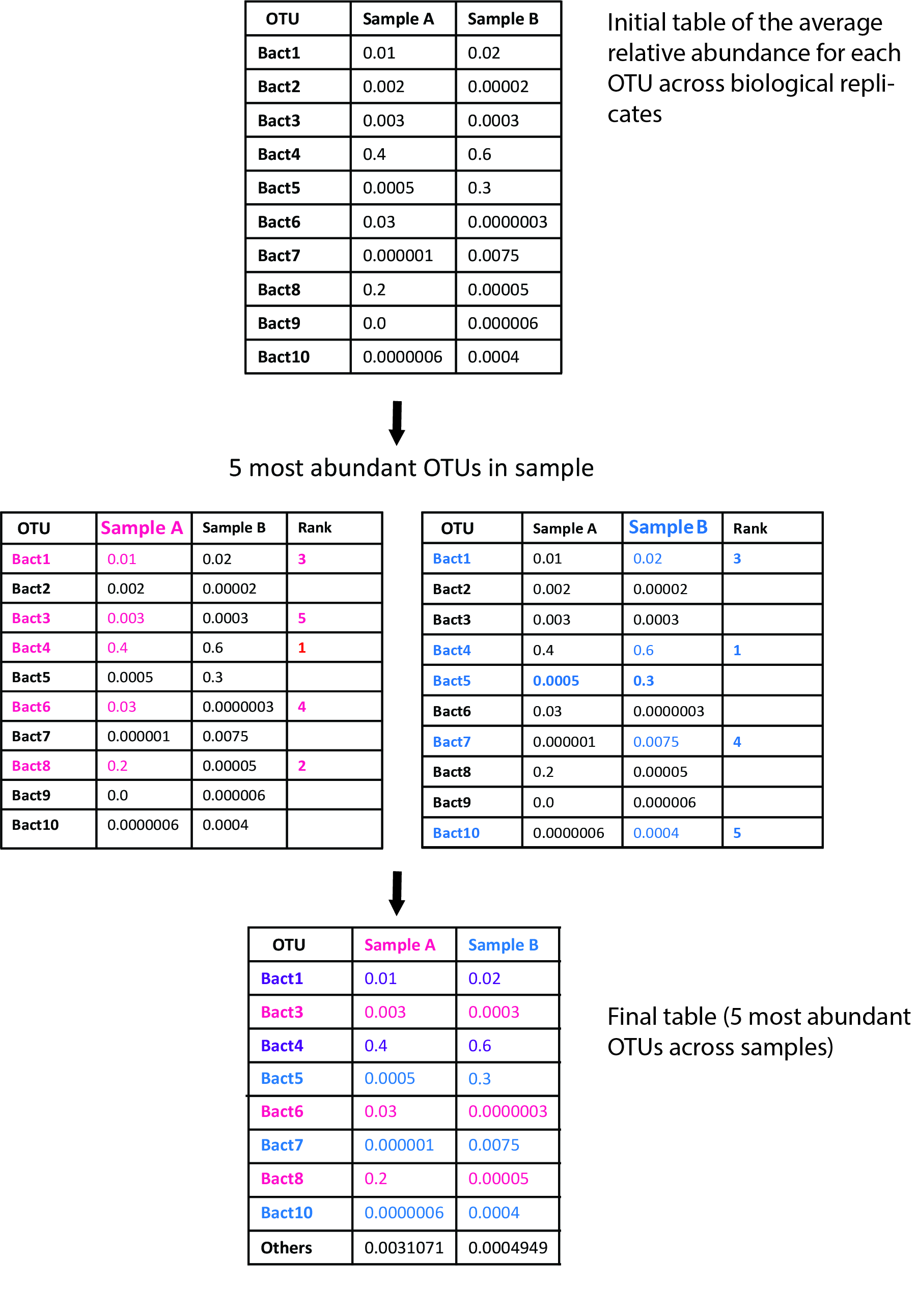

Supplement: S2 Fig — (TIF) [file pone.0239051.s002.tif]

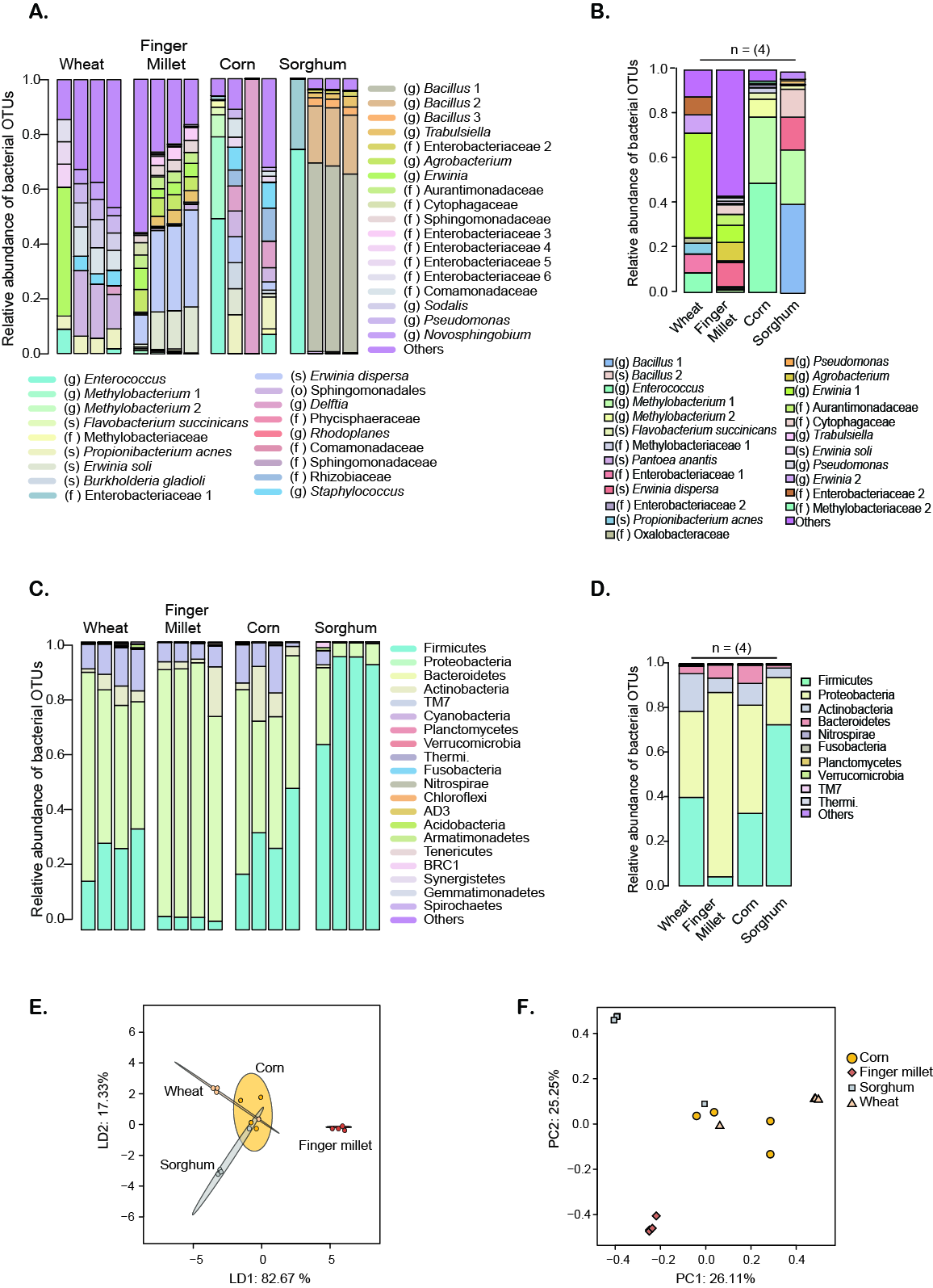

Supplement: S3 Fig — (A, C) Relative abundance of the top five dominant bacterial OTUs in individual samples of flour, classified to (A) the lowest possible taxonomic level or (C) phylum level. (B, D) Average abundance across replicates shown in panels A and C. (E) Linear Discriminant (LD) analysis and (F) Principal Coordinate Analysis (PCoA) of the complete bacterial community of flour samples; axes labels indicate % variation explained. (TIF) [file pone.0239051.s003.tif]

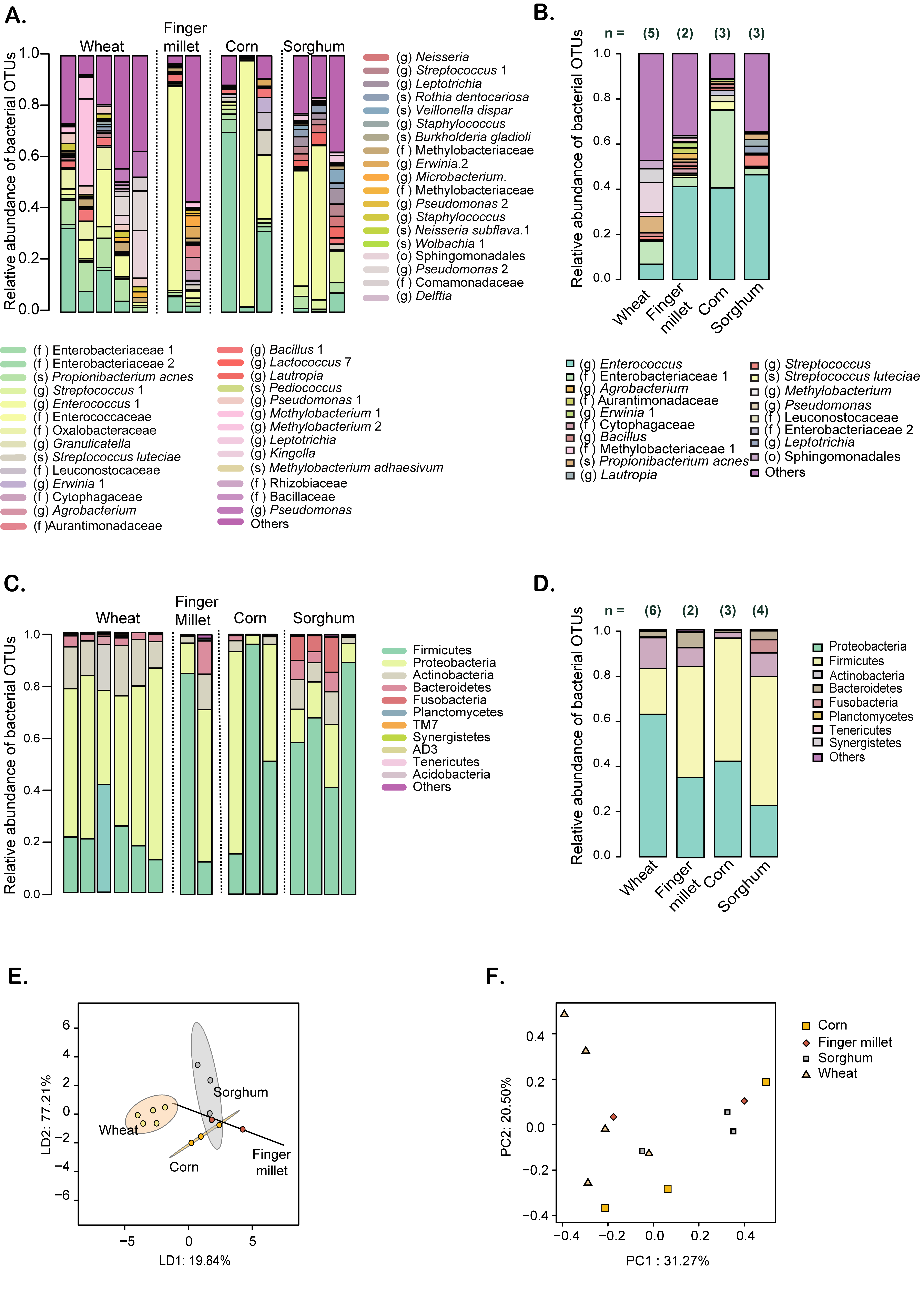

Supplement: S4 Fig — (A, C) Relative abundance of the top five dominant bacterial OTUs in individual beetle samples (isolated adult females), classified to (A) the lowest possible taxonomic level or (C) phylum level. (B, D) Average abundance across replicates shown in panels A and C. (E) Linear Discriminant (LD) analysis and (F) Principal Coordinate Analysis (PCoA) of the complete bacterial community of beetle samples; axes labels indicate % variation explained. (TIF) [file pone.0239051.s004.tif]

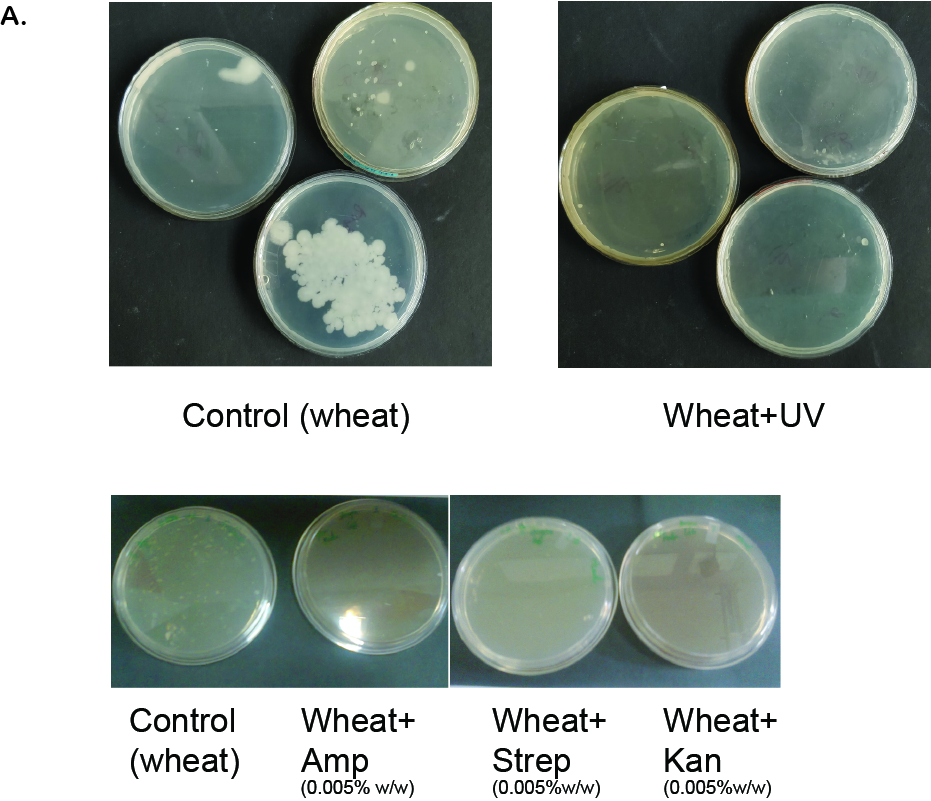

Supplement: S5 Fig — Flour samples plated onto nutrient agar plates. (TIF) [file pone.0239051.s005.tif]

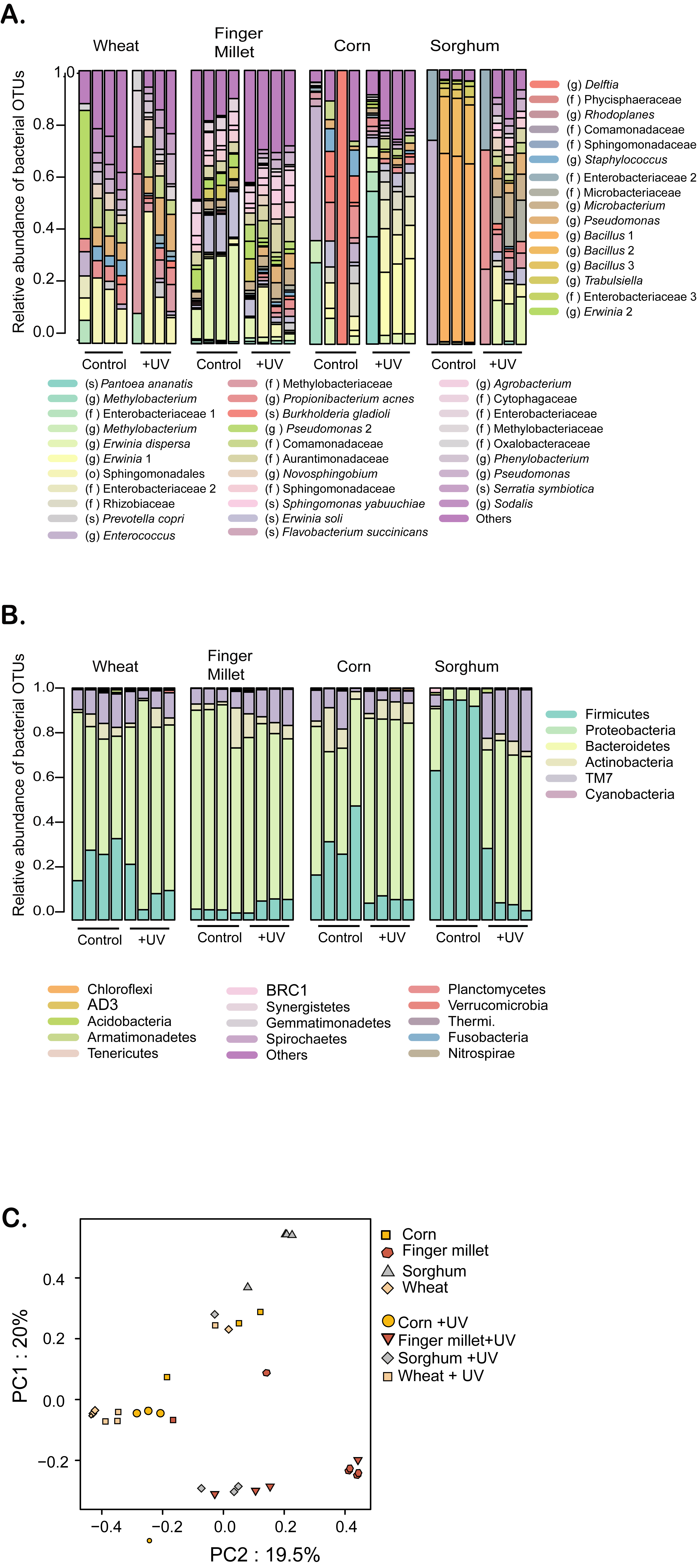

Supplement: S6 Fig — (A, B) Relative abundance of the top five dominant bacterial OTUs in individual samples of flour, classified to (A) the lowest possible taxonomic level or (B) phylum level. (C) Principal Coordinate Analysis (PCoA) of the complete bacterial community of flour samples; axes labels indicate % variation explained. (TIF) [file pone.0239051.s006.tif]

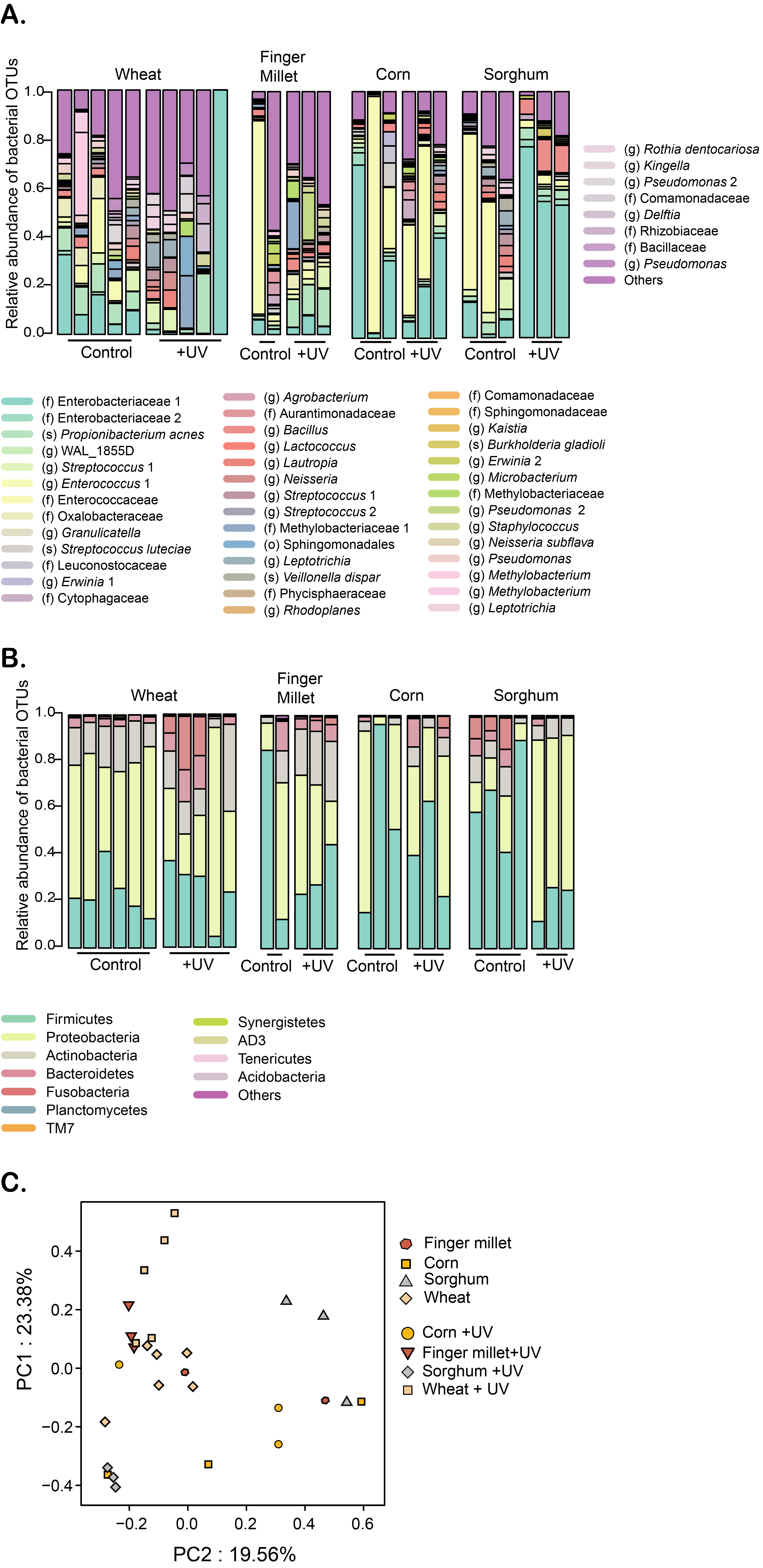

Supplement: S7 Fig — (A, B) Relative abundance of the top five dominant bacterial OTUs in beetle samples (isolated adult females reared on control vs. UV-treated flour), classified to (A) the lowest possible taxonomic level or (B) phylum level. (C) Principal Coordinate Analysis (PCoA) of the complete bacterial community of beetle samples; axes labels indicate % variation explained. (TIF) [file pone.0239051.s007.tif]

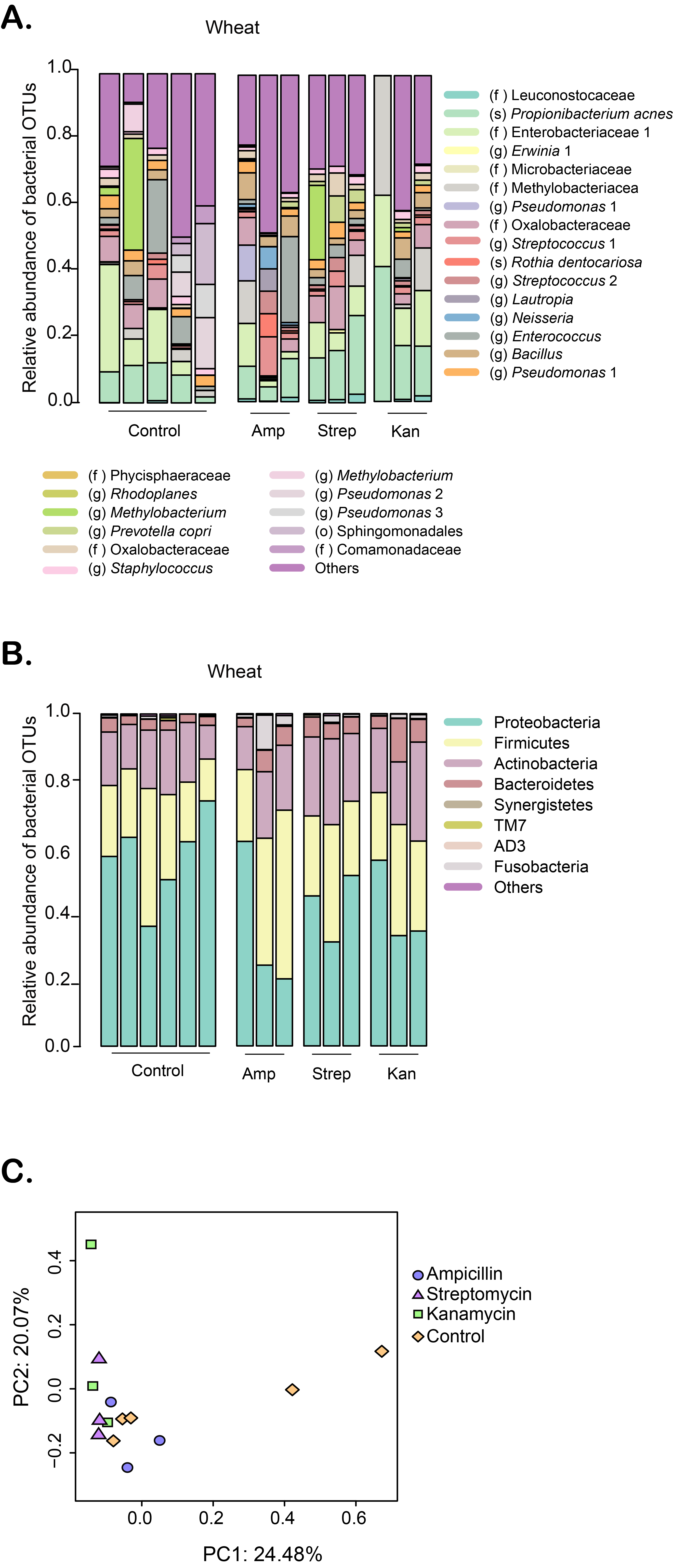

Supplement: S8 Fig — (A, B) Relative abundance of the top five dominant bacterial OTUs in beetle samples (isolated adult females reared on control vs. antibiotic-treated flour), classified to (A) the lowest possible taxonomic level or (B) phylum level. (C) Principal Coordinate Analysis (PCoA) of the complete bacterial community of beetle samples; axes labels indicate % variation explained. (TIF) [file pone.0239051.s008.tif]
