# Supplementary material for: The red flour beetle Tribolium castaneum: A model for host-microbiome interactions
Source: PLoS One. 2020 Oct 2;15(10):e0239051. doi: 10.1371/journal.pone.0239051 (PMC7531845; doi:10.1371/journal.pone.0239051)
Supplement: S1 Table — The table shows detailed statistics for comparisons of fecundity, survival, and development rate in control (untreated) or UV-treated flour (UV), with or without surface sterilization of pupae, and varying egg density during survival and development (depending on female fecundity). SS = Surface-sterilised pupae; Untreated = not surface sterilized; Conf. = confidence interval. The model specified for each analysis is indicated. Significant effects are highlighted in bold. (DOCX) [file pone.0239051.s009.docx]

**S1 Table. Summary statistics for results shown in Figure 3.** The table shows detailed statistics for comparisons of fecundity, survival, and development rate in control (untreated) or UV-treated flour (UV), with or without surface sterilization of pupae, and varying egg density during survival and development (depending on female fecundity). SS= Surface-sterilised pupae; Untreated = not surface sterilized; Conf.=confidence interval. The model specified for each analysis is indicated. Significant effects are highlighted in bold.

| **Fecundity** | | | | | | | | | | |  |
| --- | --- | --- | --- | --- | --- | --- | --- | --- | --- | --- | --- |
| ANOVA (Eggs ~ UV x Surface sterilisation x Resource) followed by pairwise Tukey’s HSD | | | | | | | | | | |  |
| Flour | Estimate | | Conf. low | | Conf. high | p value | | Effect | | | |
| Wheat | **5.2942** | | **1.1798** | | **9.408** | **0.00306** | | **Untreated pupae in control vs. UV flour** | | | |
|  | 2.8713 | | -1.306 | | 7.05 | 0.40659 | | Untreated pupae in UV flour vs. SS pupae in control flour | | | |
|  | **5.2196** | | **0.9703** | | **9.468** | **0.0057** | | **Untreated vs. SS pupae in control flour** | | | |
|  | 2.9458 | | -1.365 | | 7.257 | 0.414 | | SS pupae in control vs. UV flour | | | |
| Sorghum | 4.2857 | | -0.6809 | | 0.252 | 0.1442 | | Untreated vs. SS pupae in control flour | | | |
|  | 1.7857 | | -2.672 | | 6.243 | 0.919 | | Untreated pupae in UV vs. SS pupae in control flour | | | |
|  | 2.5000 | | -2.397 | | 7.397 | 0.7626 | | Untreated pupae in control vs. UV flour | | | |
|  | 4.6428 | | -0.3236 | | 9.609 | 0.084 | | SS pupae in control vs. UV flour | | | |
| **Survival** | | | | | | | | | | | |
| GLM ((total eggs, surviving offspring) ~ Surface sterilisation x UV; binomial error), for each resource | | | | | | | | | | |  |
| Flour | | Estimate | | Std. Error | | | p value | | Effect | | |
| Wheat | | 0.3203 | | 0.4388 | | | 0.4654 | | SS | | |
|  |  | **2.4820** | | **0.7021** | | | **0.00041** | | **UV** | | |
|  |  | -0.6778 | | 0.7995 | | | 0.39656 | | SS x UV | | |
| Sorghum | | 0.07740 | | 0.27584 | | | 0.77900 | | SS | | |
|  |  | -0.20056 | | 0.32147 | | | 0.53269 | | UV | | |
|  |  | -0.03954 | | 0.39009 | | | 0.91927 | | SS x UV | | |
| **Development** | | | | | | | | | | | |
| GLM ((total eggs, surviving pupae) ~ Surface sterilisation x UV; binomial error), for each resource | | | | | | | | | | | |
| Flour | | Estimate | | Std. Error | | | p value | | Effect |  |  |
| Wheat | | 4.445e-02 | | 3.673e-01 | | | 0.904 | | SS |  |  |
|  |  | 5.162e-01 | | 3.853e-01 | | | 0.180 | | UV |  |  |
|  |  | 1.388e-01 | | 4.439e-01 | | | 0.755 | | SS x UV |  |  |
| Sorghum | | 1.681e-02 | | 3.133e-01 | | | 0.957 | | SS |  |  |
|  |  | -1.168e-17 | | 3.761e-01 | | | 1.000 | | UV |  |  |
|  |  | -1.681e-02 | | 4.537e-01 | | | 0.970 | | SS x UV |  |  |
