# Supplementary material for: The red flour beetle Tribolium castaneum: A model for host-microbiome interactions
Source: PLoS One. 2020 Oct 2;15(10):e0239051. doi: 10.1371/journal.pone.0239051 (PMC7531845; doi:10.1371/journal.pone.0239051)
Supplement: S2 Table — (I) The table shows detailed statistics for comparisons of egg survival and development rate in control (untreated) or UV-treated flour (UV), with or without surface sterilization of pupae; and with a fixed egg density (20 eggs per replicate). SS = Surface-sterilised pupae. The model specified for each analysis is indicated. (II) The table shows detailed statistics for comparisons of egg survival in control (untreated) or UV-treated flour (UV). Significant effects are highlighted in bold. (DOCX) [file pone.0239051.s010.docx]

**S2 Table. Summary statistics for results shown in Figure 4.** (I) The table shows detailed statistics for comparisons of egg survival and development rate in control (untreated) or UV-treated flour (UV), with or without surface sterilization of pupae; and with a fixed egg density (20 eggs per replicate). SS= Surface-sterilised pupae. The model specified for each analysis is indicated. (II) The table shows detailed statistics for comparisons of egg survival in control (untreated) or UV-treated flour (UV). Significant effects are highlighted in bold.

| **I. Figure 4 A–B (consistent sibling competition)** | | | | | | | | | |
| --- | --- | --- | --- | --- | --- | --- | --- | --- | --- |
| **Survival** | | | | | | | | | |
| GLM ((total eggs, surviving offspring) ~ Surface sterilisation x UV; binomial error), for each resource | | | | | | | | | |
| Flour | | Estimate | | Std. Error | | p value | Effect | | |
| Wheat | | **-1.15947** | | **0.11466** | | **<2e-16** | **UV** | | |
|  |  | -0.319 | | 0.466 | | 0.492 | SS | | |
|  |  | 0.15202 | | 0.16425 | | 0.355 | SS x UV | | |
| Sorghum | | **0.26397** | | **0.09733** | | **0.00668** | **UV** | | |
|  |  | 0.04639 | | 0.09377 | | 0.62078 | SS | | |
|  |  | 0.08318 | | 0.13983 | | 0.55193 | SS x UV | | |
| **Development** | | | | | | | | | |
| GLM ((total eggs, surviving pupae) ~ Surface sterilisation x UV; binomial error), for each resource | | | | | | | | | |
| Flour | | Estimate | | Std. Error | | p value | Effect | | |
| Wheat | | **0.48828** | | **0.09287** | | **1.46e-07** | **UV** | | |
|  |  | 0.16834 | | 0.08790 | | 0.0555 | SS | | |
|  |  | -0.14847 | | 0.13298 | | 0.2642 | SS x UV | | |
| Sorghum | | 0.0008368 | | 0.0577652 | | 0.988 | SS | | |
|  |  | 0.0023610 | | 0.0577592 | | 0.967 | UV | | |
|  |  | -0.0011834 | | 0.0816924 | | 0.988 | SS x UV | | |
| **II. Figure 4C (no sibling competition)** | | | | | | | | |  |
| **Survival** |  | |  | |  |  | |  |  |
| GLM ((total eggs, surviving offspring) ~ UV x Resource amount; binomial error) | | | | | | | |  |  |
| Flour | Estimate | | Std. Error | | p value | Effect | |  |  |
| Wheat | **-0.8722** | | **0.1553** | | **1.96e-08** | **UV** | |  |  |
|  | **-0.8431** | | **0.1251** | | **1.62e-11** | **Resource amount** | |  |  |
|  | **0.3924** | | **0.1770** | | **0.0266** | **Resource amount x UV** | |  |  |
| **Development rate** | | | | | | | | |  |
| GLM ((total eggs, surviving pupae+adults) ~ UV x Resource amount; binomial error | | | | | | | | |  |
| Wheat | -0.01121 | | 0.07599 | | 0.8827 | UV treatment | |  |  |
|  | -0.01168 | | 0.06928 | | 0.86610 | Resource amount | |  |  |
|  | -0.03014 | | 0.09798 | | 0.75837 | Resource amount x UV | |  |  |
|  | |  | |  | |  |  | | |
