# Supplementary material for: The red flour beetle Tribolium castaneum: A model for host-microbiome interactions
Source: PLoS One. 2020 Oct 2;15(10):e0239051. doi: 10.1371/journal.pone.0239051 (PMC7531845; doi:10.1371/journal.pone.0239051)
Supplement: S3 Table — The table shows detailed statistics for comparisons of fecundity in control (untreated) or microbe-depleted flour (via UV treatment or antibiotics with the indicated concentration). Conf. = confidence interval; Amp = ampicillin. The model specified for each analysis is indicated. Significant effects are highlighted in bold. (DOCX) [file pone.0239051.s011.docx]

**S3 Table. Summary statistics for results shown in Figure 5.** The table shows detailed statistics for comparisons of fecundity in control (untreated) or microbe-depleted flour (via UV treatment or antibiotics with the indicated concentration). Conf.=confidence interval; Amp=ampicillin. The model specified for each analysis is indicated. Significant effects are highlighted in bold.

| **Fecundity** | | | | | | | | | | |  |
| --- | --- | --- | --- | --- | --- | --- | --- | --- | --- | --- | --- |
| ANOVA (Eggs ~ UV x Resource) followed by pairwise Tukey’s HSD | | | | | | | | | | |  |
| Flour | Estimate | | Conf. low | | Conf. high | | p value | | Effect | Fig |  |
| **Wheat** | **8.20** | | **3.923** | | **12.48** | | **0.000004** | | **UV** | 5A (24h) |  |
| Finger millet | -0.40 | | -4.68 | | 3.88 | | 0.9997 | | UV |  |  |
| Corn | -0.45 | | -4.80 | | 3.90 | | 0.9996 | | UV |  |  |
| Wheat vs. Finger millet | 4.067 | | -0.21 | | 8.34 | | 0.072 | | Resource |  |  |
| **Wheat vs. Corn** | **11.13** | | **6.856** | | **15.41** | | **0.000001** | | **Resource** |  |  |
| **Wheat** | **6.653** | | **2.362** | | **10.94** | | **0.0007** | | **UV** | 5A (24h) |  |
| Sorghum | -0.242 | | -4.18 | | 3.69 | | 0.998 | | UV |  |  |
| **Wheat vs. Sorghum** | **6.957** | | **2.609** | | **11.306** | | **0.000497** | | **Resource** |  |  |
| **Wheat** | **8.94** | | **2.98** | | **14.89** | | **0.0002** | | **UV** | 5A (48h) |  |
| Finger millet | 1.04 | | -4.79 | | 6.87 | | 0.9994 | | UV |  |  |
| Corn | -1.19 | | -7.02 | | 4.64 | | 0.9985 | | UV |  |  |
| Sorghum | 1.85 | | -3.99 | | 7.68 | | 0.9781 | | UV |  |  |
| Wheat vs. Finger millet | 3.51 | | -2.44 | | 9.47 | | 0.6155 | | Resource |  |  |
| **Wheat vs. Corn** | **11.94** | | **5.98** | | **17.89** | | **1.21E-07** | | **Resource** |  |  |
| **Wheat vs. Sorghum** | **6.40** | | **0.44** | | **12.35** | | **0.0255** | | **Resource** |  |  |
| **Wheat** | **23.26** | | **13.35** | | **33.181** | | **0.00000** | | **UV** | 5A (96h) |  |
| Finger millet | 4.266 | | -5.6482 | | 14.181 | | 0.887 | | UV |  |  |
| Corn | -1.90 | | -11.99 | | 8.19 | | 0.999 | | UV |  |  |
| Sorghum | 2.812 | | -5.026 | | 10.650 | | 0.954 | | UV |  |  |
| **Wheat vs. Finger millet** | **13.400** | | **3.4850** | | **23.315** | | **0.0014** | | **Resource** |  |  |
| **Wheat vs. Corn** | **32.266** | | **22.351** | | **42.182** | | **0.0000** | | **Resource** |  |  |
| **Wheat vs. Sorghum** | **16.68** | | **7.7419** | | **25.61** | | **0.000002** | | **Resource** |  |  |
| **Control vs. Amp (0.005)** | **8.80** | | **3.370** | | **14.22** | | **0.0001** | | **Antibiotic** | 5C |  |
| **Control vs. Amp (0.01)** | **9.08** | | **3.650** | | **14.50** | | **0.00009** | | **Antibiotic** |  |  |
| **Control vs. Amp (0.05)** | **8.68** | | **3.250** | | **14.11** | | **0.0002** | | **Antibiotic** |  |  |
| Chi-square | | p value | | | | df | | Effect | | Fig | |
| Kruskal-Wallis test (Eggs ~ UV) for corn (non-normal distribution) | | | | | | | | | | | |
| 1.4365 | | 0.231 | | | | 1 | | UV | | 5A (48h) | |
| Linear mixed model (Eggs ~ UV (fixed effect) x block (random effect)) | | | | | | | | | | | |
| 8.671 | | 0.0032 | | | | 1 | | UV | | 5A (7d) | |
| ANOVA (Eggs ~ antibiotics) | | | | | | | | | | | |
| Flour | | F value | | p value | | | df | Effect | | Fig | |
| **Wheat** | | **5.575** | | **0.0005** | | | **4** | **Antibiotics** | | 5C | |
| Sorghum | | 1.012 | | 0.321 | | | 1 | Antibiotics | |  |  |
| **Survival** | | | | | | | | | | | |
| Flour | | Chi-square | | p value | | | df | Effect | | Fig | |
| Chi-square test for count data (Surviving eggs ~ UV) | | | | | | | | | | | |
| **Wheat** | | **8.836** | | **0.003** | | | **1** | **UV** | | 5B | |
| Finger millet | | 1.6E-30 | | 1 | | | 1 | UV | |  |  |
| Corn | | 0.1546 | | 0.6942 | | | 1 | UV | |  |  |
| Sorghum | | 0.0655 | | 0.7979 | | | 1 | UV | |  |  |
| Chi-square test for count data (Surviving eggs ~ Resource) | | | | | | | | | | | |
| **Wheat vs. finger millet** | | **21.167** | | **4.21e-06** | | | **1** | **Resource** | | 5B | |
| **Wheat vs. corn** | | **71.209** | | **3.21e-17** | | | **1** | **Resource** | |  |  |
| **Wheat vs. sorghum** | | **11.845** | | **2.5e-08** | | | **1** | **Resource** | |  |  |
