# Supplementary material for: The red flour beetle Tribolium castaneum: A model for host-microbiome interactions
Source: PLoS One. 2020 Oct 2;15(10):e0239051. doi: 10.1371/journal.pone.0239051 (PMC7531845; doi:10.1371/journal.pone.0239051)
Supplement: S4 Table — The table shows detailed statistics for comparisons of cannibalism rates of larvae and adult females in control (untreated) or UV-treated flour (UV). Eggs were either dyed or not; and females were provided with their own eggs (self eggs) or another female’s eggs (non-self eggs) to cannibalise. The model specified for each analysis is indicated. Significant effects are highlighted in bold. (DOCX) [file pone.0239051.s012.docx]

**S4 Table. Summary statistics for results shown in Figure 7.** The table shows detailed statistics for comparisons of cannibalism rates of larvae and adult females in control (untreated) or UV-treated flour (UV). Eggs were either dyed or not; and females were provided with their own eggs (self eggs) or another female’s eggs (non-self eggs) to cannibalise. The model specified for each analysis is indicated. Significant effects are highlighted in bold.

| **Cannibalism** | | | | | |
| --- | --- | --- | --- | --- | --- |
| Life stage | Effect | Estimate | Std. Error | p value | Fig |
| GLM ((Eggs provided, Eggs remaining) ~ UV; binomial error) | | | | | |
| Larvae | **UV (dyed eggs, block I)** | **0.3788** | **0.1010** | **0.00017** |  |
|  | **UV (dyed eggs, block II)** | **-0.1994** | **0.08010** | **0.0128** | 7A |
|  | **UV (non-dyed eggs, block II)** | **-0.2822** | **0.08806** | **0.00135** |  |
| Females | **UV (self eggs)** | **-0.6320** | **0.2161** | **0.00345** | 7B |
|  | UV (non-self eggs) | 0.11817 | 0.10761 | 0.27215 |  |
| GLM ((Eggs provided, Eggs remaining) ~ Dye x UV; binomial error) | | | | | |
| Larvae (block II) | **Dyed vs. non-dyed eggs** | **-0.14210** | **0.04209** | **0.00007** | 7A |
|  | Dye x UV | 0.04139 | 0.5952 | 0.48679 |  |
