# Supplementary material for: The red flour beetle Tribolium castaneum: A model for host-microbiome interactions
Source: PLoS One. 2020 Oct 2;15(10):e0239051. doi: 10.1371/journal.pone.0239051 (PMC7531845; doi:10.1371/journal.pone.0239051)
Supplement: S5 Table — The table shows detailed statistics for comparisons of survival rates of beetles reared in untreated wheat flour (control) or UV-treated flour (UV). As pupae, some control individuals were transferred to either fresh untreated flour ((ii) in Fig 8A) or UV-treated flour ((iii) in Fig 8A). In the table, effects are indicated as shown in Fig 8. Significant effects are highlighted in bold. (DOCX) [file pone.0239051.s013.docx]

**S5 Table. Summary statistics for results shown in Figure 8.** The table shows detailed statistics for comparisons of survival rates of beetles reared in untreated wheat flour (control) or UV-treated flour (UV). As pupae, some control individuals were transferred to either fresh untreated flour ((ii) in Fig. 8A) or UV-treated flour ((iii) in Fig. 8A). In the table, effects are indicated as shown in Fig. 8. Significant effects are highlighted in bold.

| **Lifespan** | | | | |
| --- | --- | --- | --- | --- |
| Log rank test using Kaplan-Meir estimator | | | | |
| Chi-square | p value | df | Effect | Fig |
| **25.2** | **5.05e-07** | **1** | **Control vs. UV flour; (i)+(ii) vs. (iii)+(iv)** | 8A |
| 0.1 | 0.71 | 1 | Pupae moved vs. not in control flour; (i) vs. (ii) |  |
| **17.9** | **2.27e-05** | **1** | **Pupae moved into control vs. UV flour; (ii) vs. (iii)** |  |
| **37.6** | **8.6e-10** | **1** | **Control vs. UV; (i) vs. (ii)** | 8B |
| **41.9** | **4.22e-09** | **3** | **UV** | 8C |
| 1.5 | 0.218 | 1 | Infected beetles in control vs. UV |  |
| 3 | 0.083 | 1 | Sham-infected beetles in control vs. UV |  |
